# Characterization and expression profiling of cucumber kinesin genes during early fruit development: revealing the roles of kinesins in exponential cell production and enlargement in cucumber fruit

**DOI:** 10.1093/jxb/ert269

**Published:** 2013-09-10

**Authors:** Xue Yong Yang, Yan Wang, Wei Jie Jiang, Xiao Ling Liu, Xiao Meng Zhang, Hong Jun Yu, San Wen Huang, Guo Qin Liu

**Affiliations:** ^1^Institute of Vegetables and Flowers (IVF), the Chinese Academy of Agricultural Sciences (CAAS), Beijing, China; ^2^State Key Laboratory of Plant Physiology and Biochemistry, College of Biological Sciences, China Agricultural University, Beijing, China

**Keywords:** Cell division, chloroplast, cucumber, expansion, fruit, kinesin, phragmoplast, plasma membrane.

## Abstract

Rapid cell division and expansion in early fruit development are important phases for cucumber fruit yield and quality. Kinesin proteins are microtubule-based motors responsible for modulating cell division and enlargement. In this work, the candidate kinesin genes involved in rapid cell division and expansion during cucumber fruit development were investigated. The morphological and cellular changes during early fruit development were compared in four cucumber genotypes with varied fruit size. The correlation between the expression profiles of cucumber kinesin genes and cellular changes in fruit was investigated. Finally, the biochemical characteristics and subcellular localizations of three candidate kinesins were studied. The results clarified the morphological and cellular changes during early cucumber fruit development. This study found that *CsKF2*–*CsKF6* were positively correlated with rapid cell production; *CsKF1* and *CsKF7* showed a strongly positive correlation with rapid cell expansion. The results also indicated that CsKF1 localized to the plasma membrane of fast-expanding fruit cells, that CsKF2 might play a role in fruit chloroplast division, and that CsKF3 is involved in the function or formation of phragmoplasts in fruit telophase cells. The results strongly suggest that specific fruit-enriched kinesins are specialized in their functions in rapid cell division and expansion during cucumber fruit development.

## Introduction

In most plants, early fruit development can be divided into three phases: development of the ovary, cell division, and subsequent cell expansion (Gillaspy *et al.*, 1993). For cucumber fruit, the fruit weight could increase by ~200 times in 2 weeks through extremely rapid cell division [before anthesis to 5 days after anthesis (DAA)] and exponential cell enlargement (5 DAA to 14 DAA) (Marcelis and Baan Hofman-Eijer, 1993; Boonkorkaew *et al.*, 2008; Fu *et al.*, 2008, 2010). Unlike fruits that are eaten at a mature stage (such as tomato), cucumbers (*Cucumis sativus* L.), which are consumed as both a fresh product and a processed food, are typically harvested at an early phase of fruit development, namely the middle to late phase of the rapid fruit growth and ~2 weeks after anthesis (Ando *et al.*, 2012). Therefore, the rapid cell division and expansion in early fruit development are important phases for cucumber fruit production. In addition to the crucial roles in fruit size, shape, and yield, recent research has also shown that the early stage is an important determinant of fruit quality traits, such as the accumulation of sugars and organic acids and the establishment of cutin (Lemaire-Chamley *et al.*, 2005; Schlosser *et al.*, 2008; Mounet *et al.*, 2009; Ando *et al.*, 2012). A number of studies have focused on fruit ripening and post-harvest physiology; however, the mechanism of early fruit growth, especially the mechanism of rapid cell division and expansion in cucumber, is not well understood. Recently, transcriptome analyses have indicated that complicated factors are involved in early cucumber fruit growth, and cytoskeletal proteins are suggested to play important roles in this process (Ando *et al.*, 2012).

Controlling microtubule (MT) dynamics and spatial organization is a fundamental requirement of eukaryotic cell division and expansion. Kinesins constitute a superfamily of MT-dependent motor proteins and play critical roles in mitosis, meiosis, cell morphology, and the transport of vesicles and organelles (Lee and Liu, 2004; Lu *et al.*, 2005; Vanstraelen *et al.*, 2006; Hirokawa *et al.*, 2009; Li *et al.*, 2011; Zhou *et al.*, 2011). Ultimately, kinesins contribute directly or indirectly to cell division and cell growth in various tissues. In plants, several kinesins, including ATK1, ATK5, AtKRP125c, AtPAKRP1, and AtKINESIN-13A, have been reported to mediate cell mitosis and expansion in an MT-dependent manner (Lee and Liu, 2000; Chen *et al.*, 2002; Lu *et al.*, 2005; Ambrose and Cyr, 2007; Bannigan *et al.*, 2007). Recent studies have revealed that cyclin and cyclin-dependent kinases, which are important facilitators of cell production during fruit development (Malladi and Johnson, 2011), phosphorylate a mitotic kinesin protein for the appropriate onset and/or progression of cytokinesis (Sasabe *et al.*, 2011). However, it is still not known whether specific kinesin proteins are required for regulating exponential cell production and expansion during early fruit development.

Based on the interaction with cytoskeleton MTs, different kinesins have various roles in division and expansion: chromosome attachment to MTs, chromosome aggregation to the metaphase plate, sister chromatid segregation, spindle elongation, MT organization, organelle transportation for cell elongation, and control of cellulose microfibril order. Meanwhile, cell division and expansion in early cucumber fruit development have unique features, such as the rapid division and expansion speed (Marcelis and Baan Hofman-Eijer, 1993) and the precise regulation of cell proliferation and endoreduplication (Fu *et al.*, 2008, 2010). Therefore, the function of kinesin proteins in this process is expected to have unique characteristics. To understand the rapid division and expansion mechanisms of cucumber fruit, it is important to know which kinesin proteins are involved in early fruit development and what the specific functions of these proteins are. In the current study, the results indicate that kinesin proteins have specific functions in rapid cell division and during early cucumber fruit development.

## Materials and methods

### Plant materials

After seed germination, 100 plants of each cucumber (*Cucumis sativus* L.) variety, namely the ‘Chinese long’ inbred line 9930, the hybrid cultivar Zhong Nong 27, the Gherkin Nation Pickle, and 1972 B-2, were grown in pots containing mixed peat moss and vermiculite (1:1, v/v) in a greenhouse at the Institute of Vegetables and Flowers. These four varieties are of natural parthenocarpic capacity. The temperature was kept between 21 °C and 28 °C. Pest control was performed according to standard management practices.

Prior to the harvests, which were performed at –2, 0, 1, 2, 3, 5, 7, 8, 10, 12, 14, and 16 DAA, the fruits were measured for weight, length, and diameter, and were examined for changes in fruit firmness by using a ‘FHM-1 (Japan)’ penetrometer with a 1mm or 5mm diameter plunger. Each sampling and measurement used five ovaries or fruits from the eighth to 10th nodes of the main vine from five plants with three replicates. The ovaries or fruits were simultaneously sampled, frozen quickly in liquid nitrogen, and stored at –80 °C for RNA or protein analysis.

### Cell number and cell area measurement

Ovaries or fruits were sampled at 0, 3, 5, 7, 12, and 16 DAA and fixed in a mixture of 70% ethanol, formaldehyde, and acetic acid (90:5:5, v/v/v). Slices 5mm thick were cut from different parts of the fruit (outer, middle, and inner pericarp) and embedded in paraffin. Next, 8 μm thick cross-sections and longitudinal sections were prepared from the slices using a microtome. The sections were mounted, stained with haematoxylin–eosin, and photographed under a microscope (Ben-Cheikh *et al.*, 1997; Yu *et al.*, 2001). The cell number (X), cell area (A), and average cell area in a given section was calculated using Infinity capture 6.0 and Image Pro-plus5.1 software. The area of the whole fruit cross-sections and longitudinal sections (A′) was determined by measuring the ovary or fruit diameter and using the equation for the area of a circle (for a cross-section) or an ellipse (for a longitudinal section). The cell number in the whole fruit cross-sections and longitudinal sections (X′) was calculated by using the equation X/A=X′/A′. The measurements were made at five sites of each tissue for five sections from each fruit.

### Identification of kinesin genes from cucumber

All BLAST searches were conducted in the cucumber genome database by using three distinct motor domain sequences from the KHC (N-terminal motor in human), KIF2 (internal motor in mouse), and AtKCBP (C-terminal motor in *Arabidopsis*) as queries. An E-value cut-off of 1 was used, which yielded kinesins and many unrelated proteins. Domain analysis in SMART (http://smart.embl-heidelberg.de/) was then performed, and all proteins without a kinesin motor domain were eliminated. This resulted in the identification of 47 predicted kinesins in cucumber.

### RT-PCR and quantitative RT-PCR

Total RNA was isolated from various tissues of the plants using the Trizol reagent (Invitrogen) according to the manufacturer’s instructions. The RNA concentration was determined with a NanoDrop ND-2000 photospectrometer. Reverse transcription was performed with 5 μg of total RNA using M-MLV reverse transcriptase (Promega) according to the manufacturer’s instructions. The specific primers were designed for reverse transcription–PCR (RT-PCR) and quantitative real-time PCR (RT-PCR) according to the cucumber kinesin sequences (Supplementary Table S2 available at *JXB* online). Quantitative RT-PCR was performed following the protocol of the Perfect Real-time PCR kit (TaKaRa) on an iQ™5 Multicolor Real time PCR Detection system (Bio-Rad). Amplification products were visualized by SYBR Green. Aliquots of the reverse transcription reaction products were used as templates for quantitative RT-PCRs. For relative quantification, the 2^–ΔΔ*Ct*^ method (Livak and Schmittgen, 2001) was used. A cucumber *ACTIN* gene (*Csa6M484600*) was detected as an internal reference, and each experiment was repeated three times (Ando *et al.*, 2012).

### Plasmid construction

The full-length cDNA sequences of *CsKF1* (*Csa7M446860*), *CsKF2* (*Csa3M062600*), and *CsKF3* (*Csa4M002000*) are available in the cucumber genome database. Constructs were generated using sequence-specific primers (Supplementary Table S1 at *JXB* online). For protein expression *in vitro*, the coding regions for KF1-N, KF2-C, KF3-C, KF1-motor, KF2-motor, and KF3-motor were amplified by PCR and inserted into either the *p*ET28a vector or the *p*ET30a vector. For protoplast transient expression assays, the full-length cDNAs of *CsKF1* and *CsKF2* were amplified by PCR. The corresponding green fluorescent protein (GFP) fusion constructs were made by fusing the target sequences to the N-terminus of GFP, and all of the fusion constructs above were cloned into a *p*UC vector under the control of the 35S *Cauliflower mosaic virus* promoter.

### Protein expression and antibody production

His-tagged KF1-N, KF2-C, KF3-C, KF1-motor, KF2-motor, and KF3-motor fusion proteins were expressed in the *Escherichia coli* BL21 (DE3) strain, induced with 0.1mM isopropyl-β-d-thiogalactoside for 6h at 22 °C, and affinity purified using an Ni^2+^-chelating Sepharose Fast Flow (Amersham Biosciences) column following the manufacturer’s instructions. Polyclonal anti-KF1, anti-KF2, and anti-KF3 antibodies were raised in rabbits using the purified His-KF1-N, His-KF2-C, and His-KF3-C protein as the antigens. Antiserum was then affinity purified using the AminoLink Plus kit (Pierce Chemical) with immobilized antigens according to the manufacturer’s instructions.

### ATPase activity assay

MT-stimulated ATPase activity was assayed in PEM buffer (50mM PIPES, 5mM EGTA, and 1mM MgSO_4_, pH 6.9). The reaction was initiated by adding 2mM ATP to a mixture of 0–12 μM taxol-stabilized MTs and 5 μM KF1-motor or KF2-motor. The reaction lasted for 15min at 25 °C and was terminated by adding 10% trichloroacetic acid (TCA) on ice for 10min. The supernatant was collected after centrifugation at 4 °C, 20 000 *g* for 5min. The inorganic phosphate (Pi) in the supernatant was determined as described previously (LeBel *et al.*, 1978). As a control, TCA was added before the addition of ATP. The assays were repeated three times.

### Microtubule binding assay

MT co-sedimentation assays were performed in PEM buffer plus 1mM dithiothreitol (DTT) and 10 μM taxol. For each assay, 7 μM of highly purified KF1-motor or KF2-motor was incubated with 10 μM taxol-stabilized MTs for 10min at 25 °C in the absence or presence of 5mM Mg-ATP or 5mM 5′-adenylylimidodiphosphate (AMP-PNP; a non-hydrolysable ATP analogue; Sigma). The samples were then centrifuged at 100 000 *g* for 30min. The supernatants and pellets were separated, brought to equal volumes in the SDS sample buffer, analysed by SDS–PAGE, and visualized by staining the gel with Coomassie Brilliant Blue R-250.

### Immunolabelling and protoplast transient expression assays

Fruit cells (0 DAA for CsKF2, 2 DAA for CsKF3, and 5 DAA for CsKF1) were harvested at 06:00h, when cell division is rapid, and immunolabelled as described by Xu *et al.* (2009). Anti-KF1, anti-KF2, and anti-KF3 antibodies (1:500), and fluorescein isothiocyanate (FITC)-conjugated goat anti-rabbit IgG antibodies (Jackson Immuno Research Laboratories; diluted 1:200) were used to detect CsKF1, CsKF2, and CsKF3 proteins. Anti-α-tubulin monoclonal antibody (Sigma; diluted 1:500) and TRITC (tetramethylrhodamine β-isothiocyanate)-conjugated goat anti-mouse IgG (Jackson ImmunoResearch Laboratories; diluted 1:200) were used to label MTs. DNA was stained with 0.5 μg ml^–1^ DAPI (4′,6-diamidino-2-phenylindole; Sigma).

The plasmids that were used for transient expression were purified using a TIANprep Midi Plasmid Kit (TIANGEN). The fusion constructs were introduced into *Arabidopsis* mesophyll protoplasts using PEG (polyethylene glycol)-mediated transformation (Yoo *et al.*, 2007).

## Results

### Physiological changes, cell production, and cell expansion during early cucumber fruit development in 9930

**Fig. 1.**
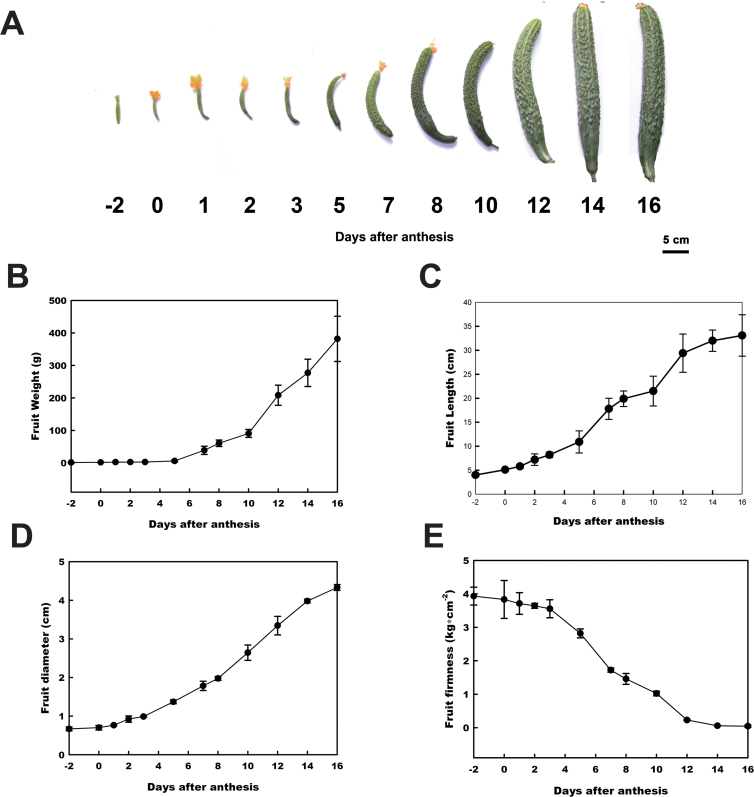
Morphological and physiological changes in 9930 during early fruit development. (A) Developing cucumber fruits at –2, 0, 1, 2, 3, 5, 7, 8, 10, 12, 14, and 16 days after anthesis (DAA). (B) Increase in the weight of 9930 fruit during early fruit development. (C) Increase in the length of 9930 fruit during early fruit development. (D) Increase in the diameter of 9930 fruit during early fruit development. (E) Decrease in the firmness of 9930 fruit during early fruit development.

The ‘Chinese long’ inbred line 9930 is a cucumber cultivar grown in northern China with an available genomic sequence (Huang *et al.*, 2009). This cucumber cultivar was grown in a greenhouse as described in the Materials and methods. It was observed that 9930 grew rapidly during the early stage, from –2 DAA to 16 DAA (Fig. 1A). There was a slight change in fruit weight between –2 DAA and 5 DAA, but the fruit weight increased sharply after 5 DAA (Fig. 1B). The fruit length and fruit diameter showed similar growth curves, initiating from –2 DAA to 3 DAA and increasing almost exponentially between 3 DAA and 16 DAA (Fig. 1C, D). The fruit firmness was relatively constant before 3 DAA and then decreased rapidly (Fig. 1E). After 16 DAA, 9930 fruit showed no significant morphological changes. These results indicated that the period from 3 DAA to 5 DAA was an obvious boundary for fast growth of the fruit of 9930.

**Fig. 2.**
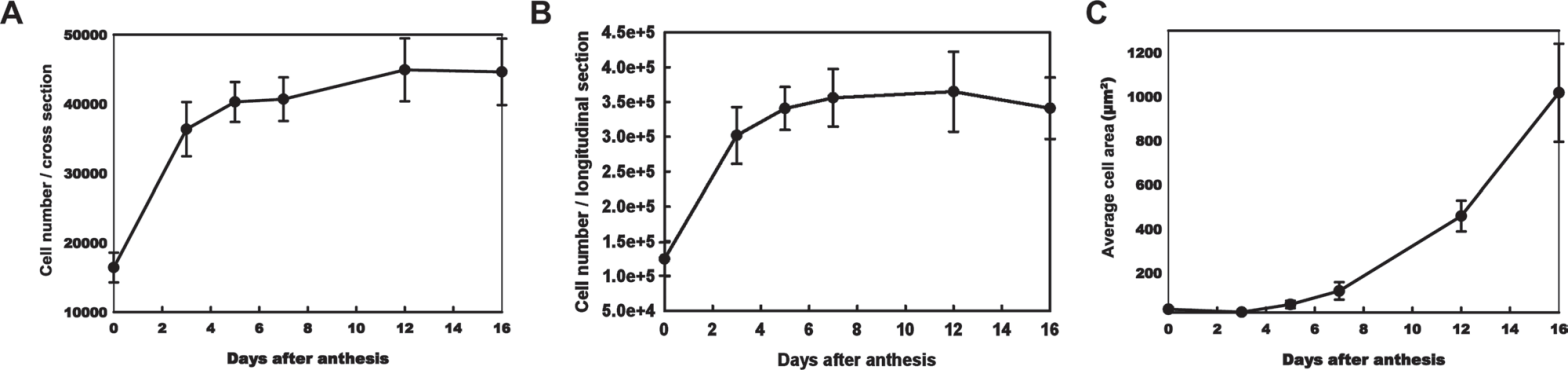
Cell production kinematics and cell expansion during early fruit development in cucumber 9930 fruit. (A) Total cell number of the cross-section in fruits during the early developing stage. (B) Total cell number of the longitudinal section in fruits during the early developing stage. (C) Average cell area of the cross-section in fruits during the early developing stage.

To clarify further the change in the cellular growth pattern during early fruit development, the cell number and average cell area were measured and calculated on the cross-section and longitudinal section of the fruit. As shown in Fig. 2, rapid increases in cell number were observed between 0 DAA and 3 DAA in both the cross-section and the longitudinal section, with an ~200% increase. The growth rate then decreased between 3 DAA and 5 DAA. Although the cell number of the cucumber fruit continued to increase from 5 DAA to 16 DAA, this occurred at a lower rate (Fig. 2A, B). The average cell area increased by <2-fold from 0 DAA to 5 DAA. However, the majority of the exponential increase in cell size (almost 30-fold) occurred between 5 DAA and 16 DAA, after the cells exited from mitotic cell production (Fig. 2C). It was also found that the changes in average cell area were similar in both the cross-section and longitudinal section directions during early 9930 fruit development (data not shown), which suggests that 9930 fruit elongation is mainly caused by an increase in cell number in the longitudinal direction rather than increased cell expansion in the longitudinal direction.

Together, the above data indicated that early fruit growth was closely associated with cell production before 3 DAA and that the cell division rate decreased between 3 DAA and 5 DAA. At this time, cell expansion was initiated, and fruit growth was primarily driven by cell expansion after 5 DAA.

### Identification of cucumber kinesin genes

To investigate the function of cucumber kinesin genes during early fruit development, 47 genes were identified in the cucumber genome by using the conserved kinesin motor domains of human, mouse, and *Arabidopsis* as BLAST queries in the cucumber genome database (http://www.icugi.org/cgi-bin/ICuGI/). All of the 47 kinesin genes have corresponding homologues in *Arabidopsis* (Supplementary Table S3 at *JXB* online). According to the phylogenetic analysis of the cucumber kinesins and the previously recognized family, all predicted cucumber kinesins were found to be distributed across all kinesin families except families 2, 3, 9, and 11, which was consistent with a previous report in the land plant (Zhu and Dixit, 2011). The results also showed that all of these cucumber families were smaller than those in *Arabidopsis*, with the exception of the kinesin-13 family. Cucumber has three members in this family, whereas *Arabidopsis* only has two (Supplementary Fig. S1). Moreover, the expression of 47 cucumber kinesin genes during vegetative (root, stem, tendril, true leaf, cotyledon, and hypocotyl) and reproductive development (dry seed, geminated seed, male flower, female flower, ovary, 0 DAA fruit, 2 DAA fruit, and 10 DAA fruit), measured by using RT-PCR, indicated that all of these predicted kinesins were expressed genes and were differentially expressed in various tissues (Supplementary Fig. S2).

### Kinesin gene expression patterns in early cucumber fruit development

**Fig. 3.**
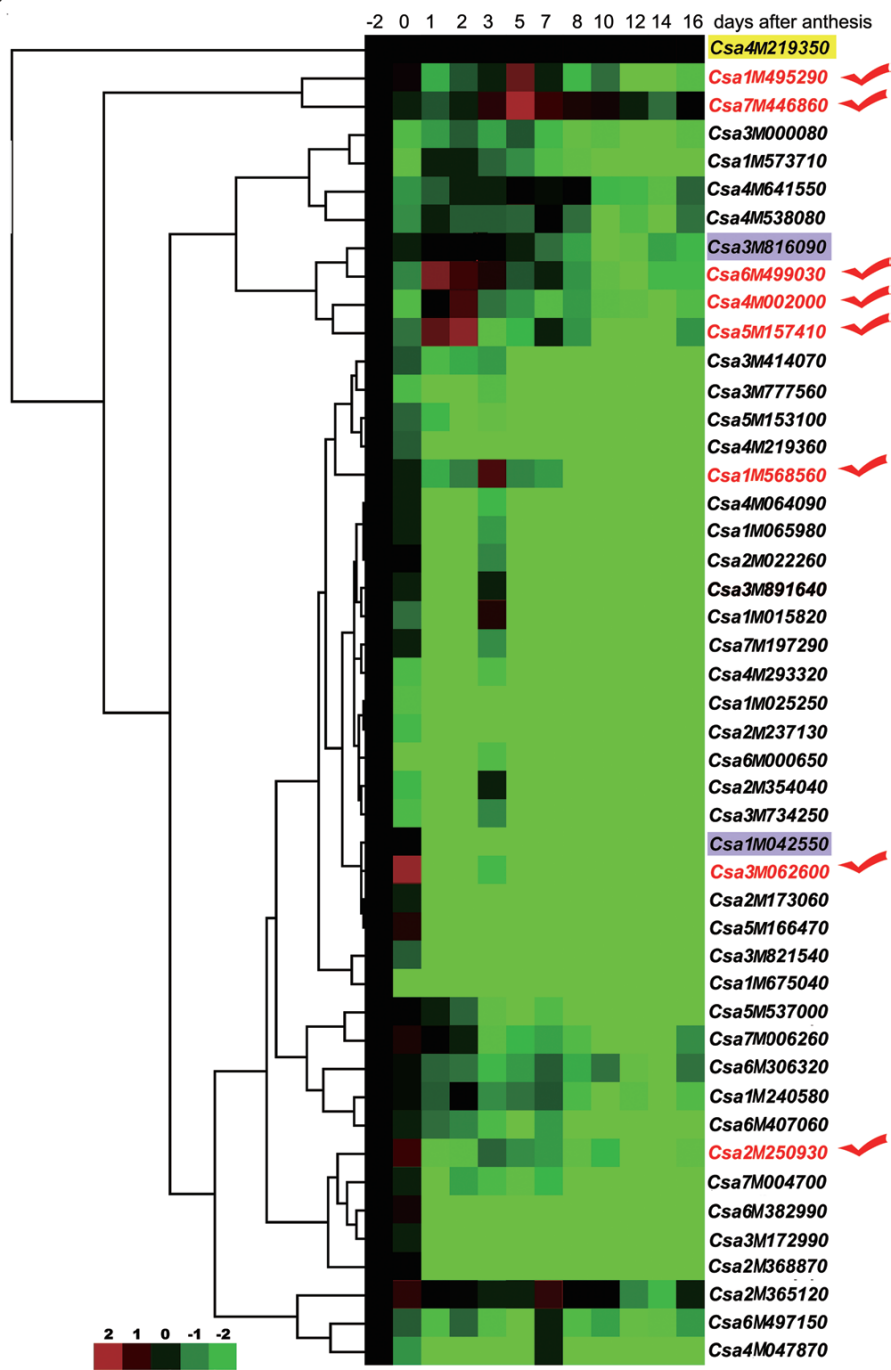
Expression profiles of kinesin family genes during early fruit development in cucumber by hierarchical clustering. Log_2_-transformed data were used for the cluster analysis (*n*=3). Expression data for a given gene are shown relative to the expression at –2 DAA. The inset shows the colour legend used in the cluster representation (log_2_ ratios). *CsACTIN* genes were used for normalization of quantitative RT-PCR results.

To find the potential candidate kinesin proteins that are involved in early cucumber fruit development, actively transcribed genes were measured using quantitative RT-PCR. The quantitative changes of kinesin gene expression were compared with changes in cell production and expansion, and it was possible to observe the expression patterns of kinesin genes in early 9930 fruit development (Fig 3; Supplementary Fig. S3 at *JXB* online). As shown in Fig. 3, hierarchical clustering gave a more visual analysis of kinesin gene expression. All of the cucumber kinesin genes, except *Cs4g219350*, showed significant changes in expression during early fruit development (from –2 DAA to 16 DAA). Further analysis indicated that 10 kinesin genes (labelled in red and blue) showed peak expression levels in the rapid cell division phase (before 3 DAA), while two kinesin genes showed peak expression levels during the rapid cell expansion (after 5 DAA) (Fig. 3; Supplementary Table S4). Eight of these kinesin genes that showed at least a 4-fold up-regulation were selected for further study (labelled by the red ticks; Fig. 3). *Csa6M499030*, *Csa4M002000*, *Csa2M250930*, *Csa5M157410*, *Csa3M062600*, and *Csa1M568560* displayed enhanced levels of expression during the rapid cell production phase, between –2 DAA and 3 DAA, while both *Csa1M495290* and *Csa7M446860* are typically characterized by the specific up-regulation at the initiation of exponential cell expansion, ~5 DAA during early fruit development.

### Physiological and cellular characterization of fruit in four cucumber varieties with different fruit size

Because fruit cell number and cell size are important determinants of fruit size, it was decided to clarify further the relationship between the eight selected kinesin genes and rapid cell division or expansion. Therefore, the gene expression profiles were investigated in three other cucumber varieties with varied fruit size during early fruit development. Two of the cucumber varieties, 1972 B-2 and National Pickle, are pickling cucumbers with relatively smaller fruit, while the third variety, Zhong Nong 27, has relatively larger fruit and is a hybrid variety found in northern China.

**Fig. 4.**
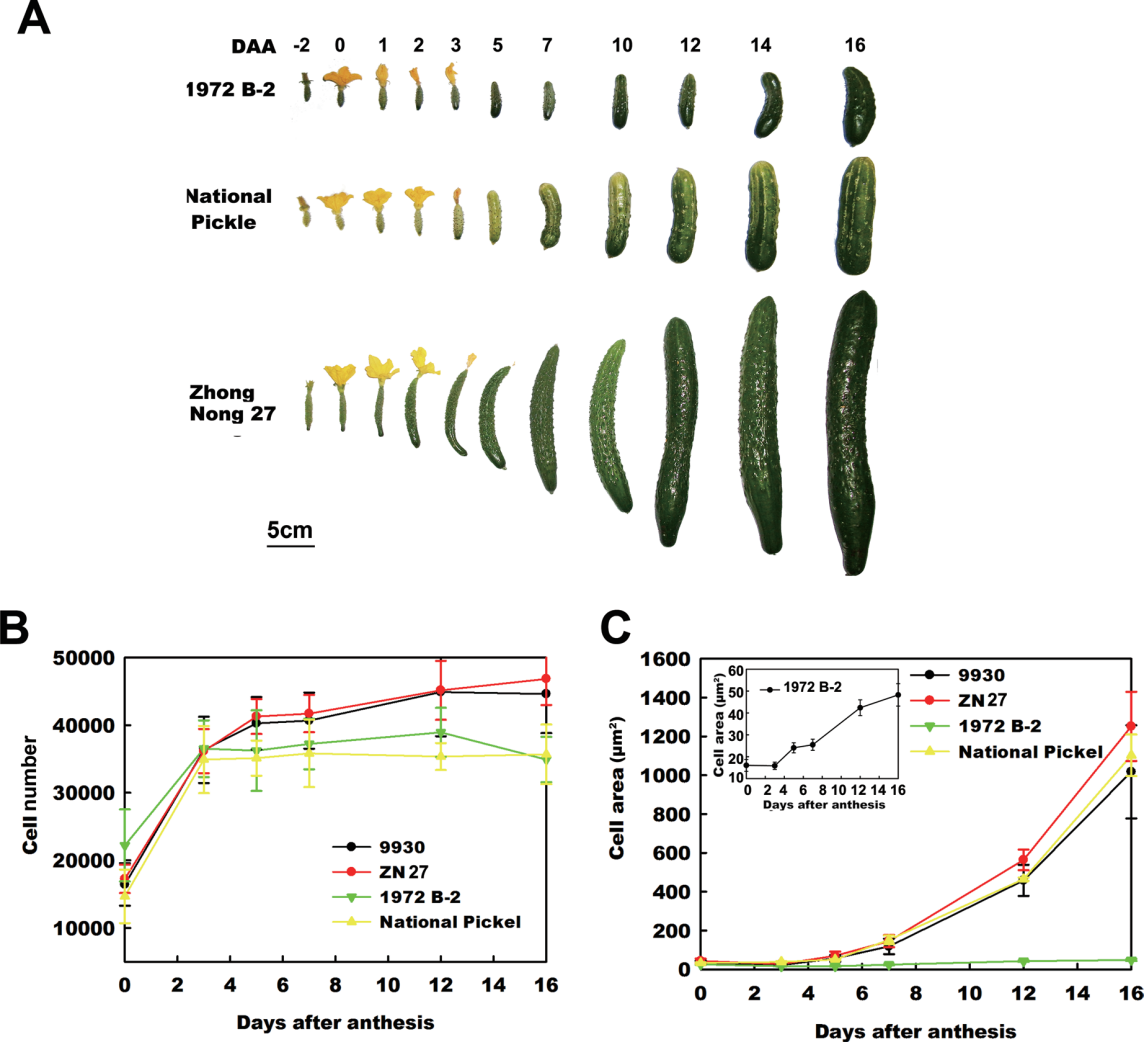
Comparative analysis of morphological and cellular changes in the four varieties during early fruit development. (A) Developing fruits at –2, 0, 1, 2, 3, 5, 7, 8, 10, 12, 14, and 16 DAA in 1972 B-2, National Pickle, and Zhong Nong 27. (B) Comparative analysis of cell production during early fruit development in 1972 B-2, National Pickle, 9930, and Zhong Nong 27. (C) Comparative analysis of cell expansion during early fruit development in 1972 B-2, National Pickle, 9930, and Zhong Nong 27.

The morphological and cellular changes in the three varieties were characterized during early fruit development. As shown in Fig. 4A, 1972 B-2 produced the smallest fruit, National Pickle produced a relatively larger fruit, and Zhong Nong 27 produced the largest fruit during early fruit development (from –2 DAA to 16 DAA). After 16 DAA, these varieties showed little change in fruit size. From the largest to the smallest, the cucumber varieties rank in fruit size as follows: Zhong Nong 27 > 9930 > National Pickle > 1972 B-2 (Figs 1, 4). Similar to the observations of 9930, it was found that the stage from 3 DAA to 5 DAA was also a period of transition in fruit growth for 1972 B-2, National Pickle, and Zhong Nong 27 (Supplementary Fig. S4 at *JXB* online). However, the growth rates of fruit weight, length, and diameter were much higher in the variety with larger fruit (Supplementary Fig. S4).

To understand the cellular changes in the four cucumber varieties during early fruit development, the cell number and average cell area of the total cross-section of the fruit were observed and compared. Figure 4B shows that similarly to 9930, Zhong Nong 27 exhibits an exponential increase in cell number between 0 DAA and 3 DAA, resulting in a 200% increase in cell number. Between 3 DAA and 5 DAA, there was a small increase in the cell number, and the cell number increased very slowly from 5 DAA to 16 DAA (Fig. 4B). Both 1972 B-2 and National Pickle also showed very rapid increases in cell number by almost 200% between 0 DAA and 3 DAA; however, from 3 DAA to 16 DAA, the cell number of these varieties exhibited constant growth curves (Fig. 4B). The order of the final cell number at 16 DAA is 9930 ≈ Zhong Nong 27 >> 1972 B-2 ≈ National Pickle (A ≈ B >> C ≈ D; Fig. 4B; Table 1). Interestingly, three varieties, 9930, Zhong Nong 27, and National Pickle, showed a rapid increase in the average cell area from 5 DAA to 16 DAA, resulting in a 30-fold increase. However, the cell area of 1972 B-2 only increased by ~3-fold, which could explain why 1972 B-2 bears the smallest fruit (Fig. 4C). The order of the final average cell area at 16 DAA is Zhong Nong 27 > 9930 ≈ National Pickle >> 1972 B-2 (B > A ≈ D >> C; Fig. 4C; Table 1). These results indicate that both cell numbers and cell area correlate strongly with fruit size.

**Table 1.** Cell changes in four cucumber varieties during early fruit development

	Cell number	Cell area
Change rules	Increase between –2 DAA and 3 DAA	Increase between 5 DAA and 16 DAA
Difference in four varieties	A ≈ B >> C ≈ D	B > A ≈ D >> C

A represents ‘9930’; B represents ‘Zhong Nong 27’; C represents ‘1972 B-2’; and D represents ‘National Pickle’.

### Identification of seven candidate kinesins involved in rapid fruit cell division and expansion

**Fig. 5.**
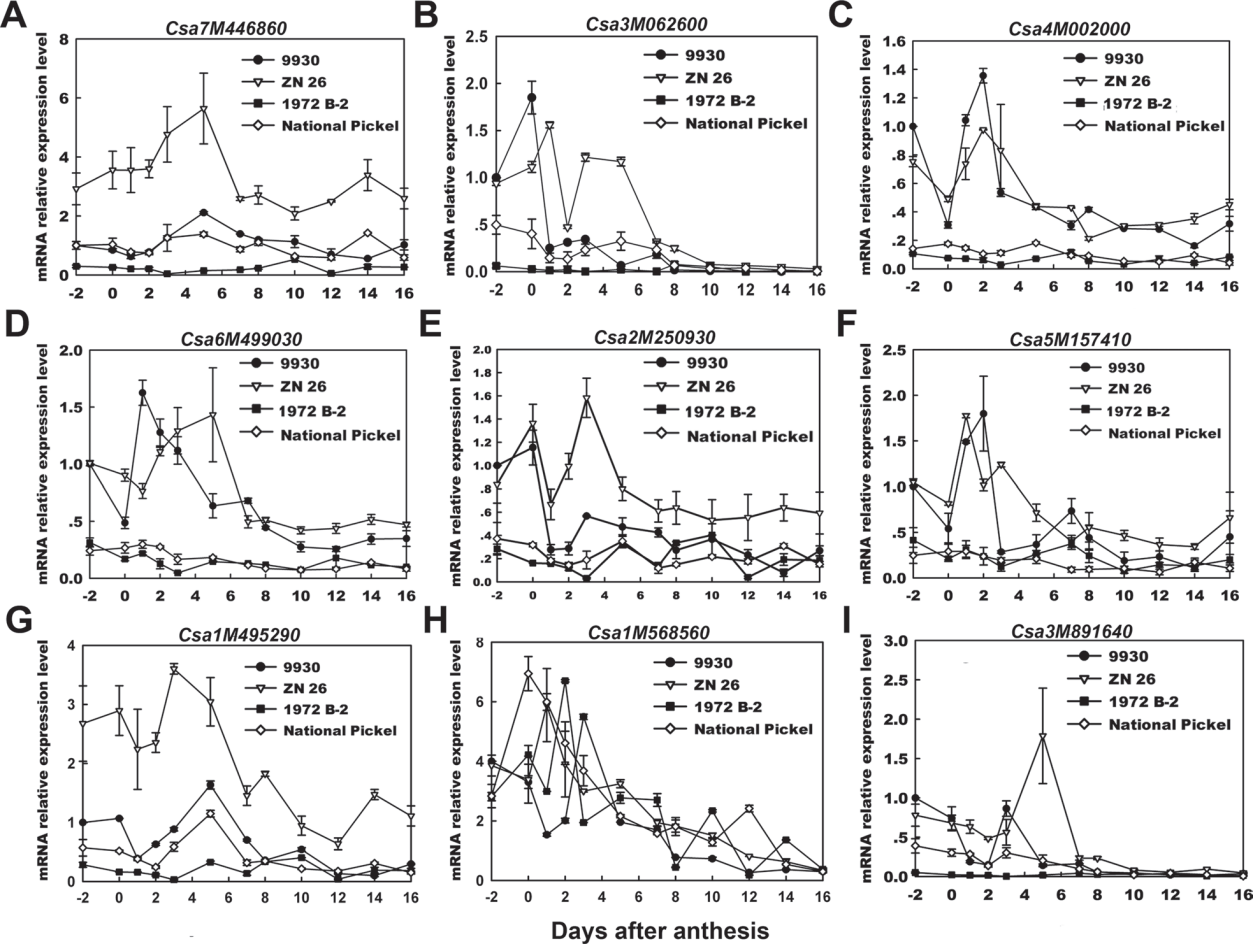
Comparative analysis of expression patterns of eight candidate kinesin genes, *Csa7M446860* (A), *Csa3M062600* (B), *Csa4M002000* (C), *Csa6M499030* (D), *Csa2M250930* (E), *Csa5M157410* (F), *Csa1M495290* (G), and *Csa1M568560* (H), and one control kinesin gene *Csa3M891640* (I), during early fruit development in 1972 B-2, National Pickle, 9930, and Zhong Nong 27.

The expression of the eight predicted kinesins was further analysed in these four characterized varieties during early fruit development. As expected, seven of the eight kinesin genes, *Csa7M446860*, *Csa3M062600*, *Csa4M002000*, *Csa6M499030*, *Csa2M250930*, *Csa5M157410*, and *Csa1M495290*, showed different expression levels that correlated with the differences in cell number or expansion among the four cucumber varieties (Fig. 5). According to the result from quantitative trait loci (QTL) mapping of domestication-related traits, these seven candidate kinesin genes were also identified to be the potential trait genes selected during cucumber domestication (data not shown). *Csa3M062600*, *Csa4M002000*, *Csa6M499030*, *Csa2M250930*, and *Csa5M157410* were up-regulated before 3 DAA, and the expression levels of these genes correlated with the difference in cell numbers in these four varieties (Fig. 5B–F; Table 2). Additionally, *Csa7M446860* and *Csa1M495290* were up-regulated after 5 DAA, and the expression levels of these genes correlated with the order of cell area in these four varieties (Fig. 5A, G; Table 2). However, the expression levels of the remaining kinesin gene, *Csa1M568560*, showed no significant difference among these four varieties (Fig. 5H). *Csa3M891640*, an unrelated gene, was used as a control (Fig. 5I).

**Table 2.** Expression patterns of predicted kinesin genes in the four varieties during early fruit development

Gene ID	Peak expression	Differences in expression level in the four varieties
*Csa7M446860*	5 DAA	B > A ≈ D >> C
*Csa3M062600*	0 DAA	A ≈ B >> C ≈ D
*Csa4M002000*	2 DAA	A ≈ B >> C ≈ D
*Csa6M499030*	2 DAA	A ≈ B >> C ≈ D
*Csa2M250930*	0 DAA	A ≈ B >> C ≈ D
*Csa5M157410*	1 DAA	A ≈ B >> C ≈ D
*Csa1M495290*	5 DAA	B > A ≈ D >> C
*Csa1M568560*	0–4 DAA	A ≈ B ≈ C ≈ D
*Csa3M891640*	0–5 DAA	B > A ≈ C > D

A represents ‘9930’; B represents ‘Zhong Nong 27’; C represents ‘1972 B-2’; and D represents ‘National Pickle’.

These results strongly suggest that these seven candidate kinesin genes are involved in early cucumber fruit development. *Csa3M062600*, *Csa4M002000*, *Csa6M499030*, *Csa2M250930*, and *Csa5M157410* play roles in rapid cell production, while *Csa7M446860* and *Csa1M495290* function in exponential cell expansion.

### Identification of *CsKF1*, *CsKF2*, and *CsKF3*

**Fig. 6.**
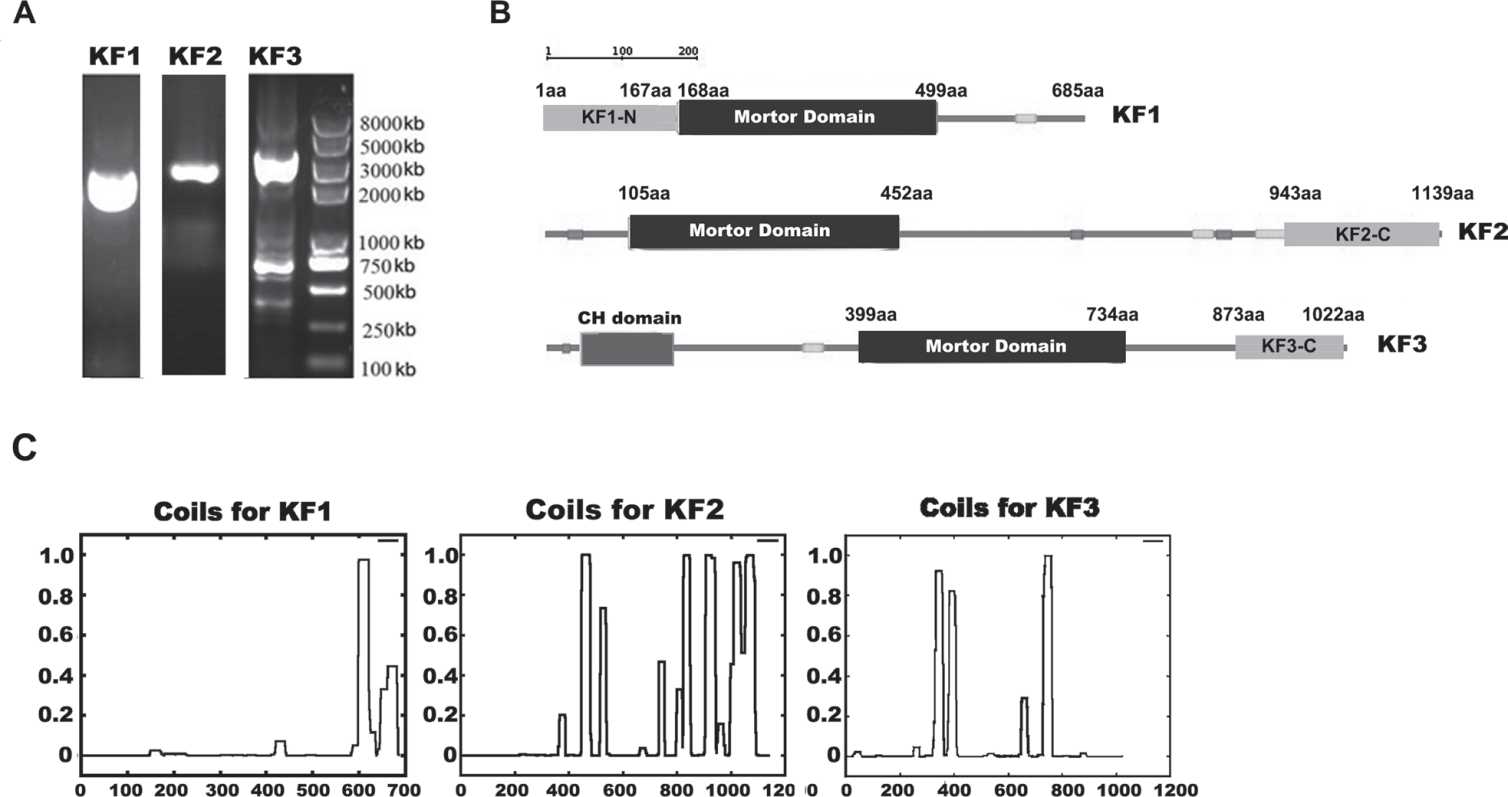
Cloning and identification of *CsKF1*, *CsKF2*, and *CsKF3* genes. (A) The full-length CDS of *CsKF1*, *CsKF2*, and *CsKF3* were obtained by RT-PCR using specific primers designed according to the cucumber genome database. (B) Schematic diagram for the domain organization of CsKF1, CsKF2, and CsKF3 analysed in SMART (http://smart.embl-heidelberg.de/), indicating that these kinesins have putative kinesin motor domains in the central region. The truncated peptides CsKF1-N, CsKF2-C, and CsKF3-C for preparation of polyclonal antibodies are also indicated in the diagram. (C) Prediction of coiled-coils in CsKF1, CsKF2 and CsKF3 by COILS (http://www.ch.embnet.org/software/COILS_form.html). The *x*-axis represents the position of amino acid residues, and the *y*-axis represents the probability of the formation of coiled-coils. A probability value >0.5 indicates that the corresponding region most probably forms a coiled-coil.

For a more concise description in the following research, the seven candidate kinesin genes were then designated as *CsKF1*, *CsKF2*, *CsKF3*, *CsKF4*, *CsKF5*, *CsKF6*, and *CsKF7* (KF is an abbreviation for ‘kinesin in fruit’), as indicated in Table 3. The corresponding *Arabidopsis* homologues, families, and predicted functions are also indicated in Table 3. The functions of all seven candidate kinesins *CsKF1*–*CsKF7* in cucumber fruit need further investigation. In this work, *CsKF1* (*Csa7M446860*), *CsKF2* (*Csa3M062600*), and *CsKF3* (*Csa4M002000*), whose *Arabidopsis* homologues have unknown functions, were first selected for further study. The full-length CDS (coding sequences) of *CsKF1* (2058bp), *CsKF2* (3420bp), and *CsKF3* (3066bp) were obtained by using the primers that were specifically designed according to the gene sequences found in the Cucurbit Genomics Database (Fig. 6A). The deduced CsKF1 protein has a calculated molecular mass of 77kDa and a pI of 6.4, the CsKF2 protein consists of 1139 amino acids with a calculated molecular mass of 128kDa and a pI of 6.2, and the CsKF3 protein consists of 1022 amino acids with a calculated molecular mass of 112kDa and a pI of 8.3. Schematic structure analyses of CsKF1, CsKF2, and CsKF3 indicate that these kinesins have conserved motor domains (Fig. 6B). Phylogenetic analyses with other representative kinesins in *Arabidopsis* and rice were performed to investigate how these polypeptides relate to other kinesins. CsKF1 belongs to the kinesin-13 family, CsKF2 belongs to the kinesin-12 family, and CsKF3 belongs to the kinesin-14 family (Supplementary Fig. S1 at *JXB* online). The secondary structure prediction showed the localization of coiled-coil regions in these three kinesins, which are required for oligomerization of kinesin proteins in their native forms (Fig. 6C). RT-PCR expression assays demonstrated that *CsKF1* is expressed in all examined tissues but was enriched in developed fruit (Supplementary Fig. S2). *CsKF2* and *CsKF3* were expressed in all tissues except the seed, and were also enriched in developed fruit (Supplementary Fig. S2).

**Table 3.** List of seven candidate cucumber kinesins involved in rapid cell division and expansion

Gene ID	Name	*Arabidopsis* homologue	Family	Function of *Arabidopsis* homologue	Predicted function in cucumber fruit
*Csa7M446860*	*CsKF1*	KINESIN-13B	Kinesin-13	A mitosis-specific kinesin localizes in plasma membrane with unknown function	Rapid cell expansion
*Csa3M062600*	*CsKF2*	AT3G20150	Kinesin-12	Unknown	Rapid cell division
*Csa4M002000*	*CsKF3*	AT2G47500	Kinesin-14	Unknown	Rapid cell division
*Csa6M499030*	*CsKF4*	KCA1/KAC1	Kinesin-14	For cytokenesis and actin-based chloroplast movement	Rapid cell division
*Csa2M250930*	*CsKF5*	MKRP2	Kinesin-7	Predicted to work in mitochondria	Rapid cell division
*Csa5M157410*	*CsKF6*	ARK3	Ungrouped	Accumulates at the pre-prophase band (PPB) in a cell cycle	Rapid cell division
*Csa1M495290*	*CsKF7*	AT3G45850	Kinesin-5	A mitosis-specific kinesin localizes in plasma membrane with unknown function	Rapid cell expansion

The final seven candidate kinesins predicted to be involved in early fruit development were designated to *CsKF1*, *CsKF2*, *CsKF3*, *CsKF4*, *CsKF5*, *CsKF6*, and *CsKF7*, and the corresponding *Arabidopsis* homologues, families, and predicted functions are indicated.

### Biochemical identification of the CsKF1 motor and CsKF2 motor

**Fig. 7.**
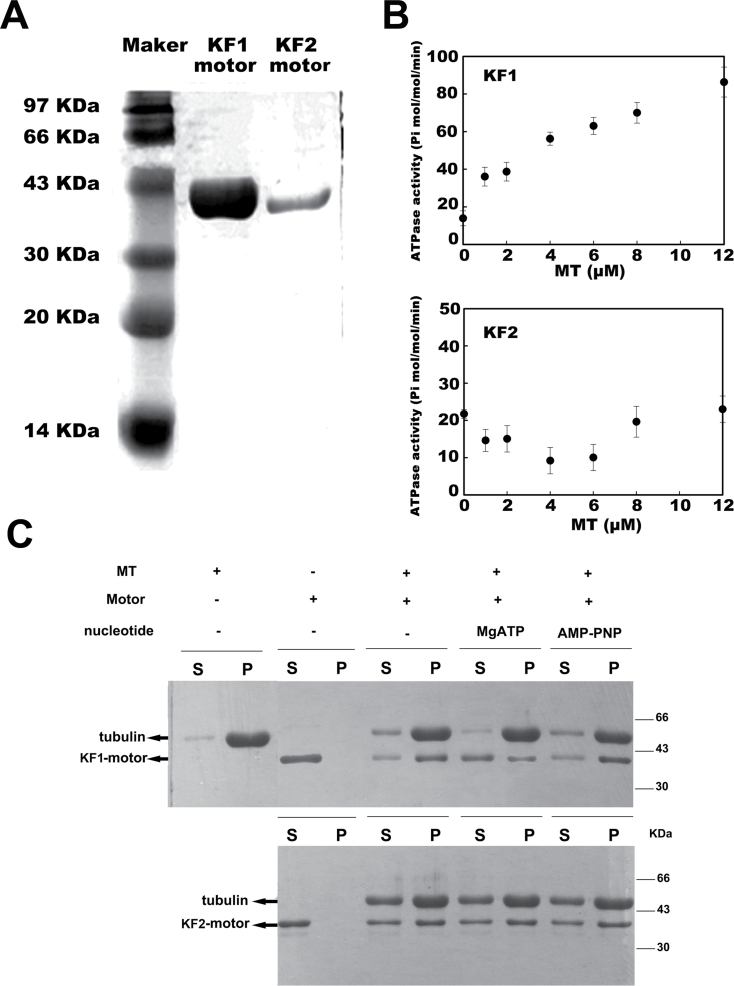
*In vitro* ATPase activities and microtubule (MT) binding abilities of CsKF1 and CsKF2 motors. (A) Prokaryotic expression and affinity purification of His-tagged CsKF1 and CsKF2 motor domain fusion proteins. (B) MT concentration dependence of CsKF1-motor and CsKF2-motor ATPase activities. (C) MT binding assay. The resulting supernatant (S) and pellet (P) were analysed by SDS–PAGE and visualized by Coomassie blue staining. For each assay, 7 μM motor proteins were used for MT binding in the absence or presence of 5mM MgATP or AMP-PNP.

Kinesins are molecular motors that hydrolyse ATP as they move along MTs, and these proteins exhibit MT-stimulated ATPase activity (Vale and Fletterick, 1997). To examine whether the motor domains of CsKF1, CsKF2, and CsKF3 have traditional biochemical characteristics (MT binding ability and MT-stimulated ATPase activity), a histidine-tagged CsKF1 motor domain (from amino acid 168 to 499) and a histidine-tagged CsKF2 motor domain (from amino acid 105 to 452) were solubly expressed in *E. coli* and purified from the cell extract by Ni-NTA agarose affinity chromatography as 40kDa polypeptides (Fig. 7A). Unfortunately, the soluble-expressed motor domain of CsKF3 could not be obtained in *E. coli* under several expression conditions. This could have been because two short coiled-coil domains located in the predicted motor of CsKF3 reduce the solubility of this protein (Fig. 6D).

Figure 7B shows the results of an MT-stimulated ATPase activity assay. In the absence of MTs, the basal steady-state ATPase activity of the CsKF1 motor and the CsKF2 motor was 13.89±3.97 Pi mol min^–1^ mol^–1^ and 21.72±1.18 Pi mol min^–1^ mol^–1^, respectively (Fig. 7B). Moreover, the ATPase activity of the CsKF1 motor was triggered by MTs in a concentration-dependent manner, whereas, surprisingly, the ATPase activity of the CsKF2 motor was independent of the MT concentration. MTs alone showed no detectable ATPase activity (Fig. 7B).

These fusion proteins were further used in MT co-sedimentation experiments. In the absence of an exogenous nucleotide, the His-CsKF1-motor largely sedimented with the MT pellet. In the presence of ATP, >50% of the fusion protein was found in the supernatant rather than in the MT pellet. When ATP was replaced by the non-hydrolysable ATP analogue adenylyl imidodiphosphate (AMPPNP), the fusion protein appeared almost exclusively in the MT pellet (Fig. 7C). However, the His-CsKF2-motor co-sedimented with the MT pellet regardless of the presence of exogenous nucleotides (Fig. 7C).

These results indicate that His-CsKF1-motor has an MT-stimulated ATPase activity and binds to MTs in an ATP-dependent manner, which is consistent with the traditional characterization of a kinesin protein. However, the ATPase activity of the His-CsKF2-motor is independent of MT concentration, and this protein binds to MTs in a nucleotide-independent manner.

### CsKF1, CsKF2, and CsKF3 localizations in cucumber fruit cells and *Arabidopsis* protoplasts

**Fig. 8.**
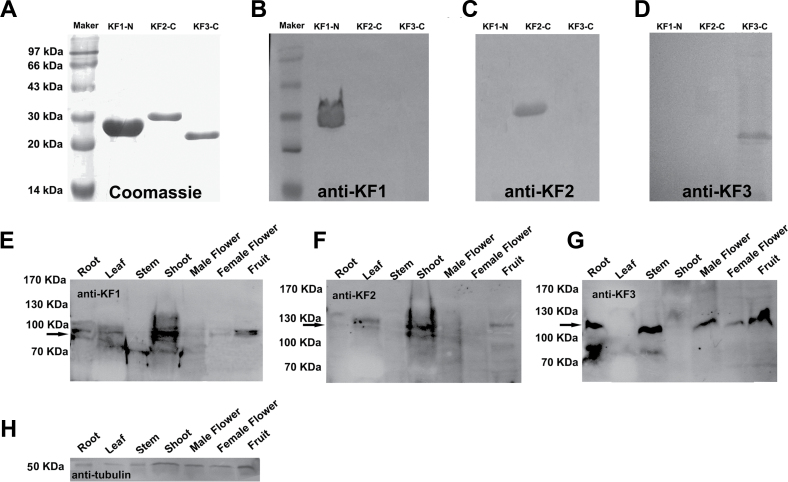
Preparation of CsKF1, CsKF2, and CsKF3 antibodies. Prokaryotic expression and affinity purification of His-tagged CsKF1-N, CsKF2-C, and CsKF3-C fusion proteins used as antigen proteins (A). The polyclonal antibodies could specifically identify the corresponding antigen peptides KF1-N (B), KF2-C (C), and KF3-C (D). Western blot analysis of CsKF1, CsKF2, and CsKF3 in proteins of root, leaf, stem, shoot, male flower, female flower, and fruit. An ~80kDa protein from cucumber root, leaf, shoot, and fruit reacted with the anti-KF1 antibody (E); an ~125kDa protein from cucumber leaf, shoot, and fruit reacted with the anti-KF2 antibody (F); and an ~110kDa protein from cucumber root, stem, flower, and fruit was recognized by the anti-KF3 antibody (G). An anti-α-tubulin antibody was used in a positive control experiment, and the 50kDa protein band was revealed in all samples (H).

To gain insight into the functions of CsKF1, CsKF2, and CsKF3 in early fruit development, the intracellular localization pattern was determined in fruit cells via immunofluorescence labelling. Antibodies were raised against the affinity-purified His-CsKF1-N-terminus (KF1-N; amino acids 1–167), His-CsKF2-C-terminus (KF2-C; amino acids 943–1139), and the His-CsKF3-C-terminus (KF3-C; amino acids 873–1022) (Fig. 8A). Affinity-purified anti-KF1-N, anti-KF2-C, and anti-KF3-C antibodies could specifically recognize their corresponding prokaryotic-expressed His-tagged antigens, but did not recognize the two other proteins (Fig. 8B–D). The anti-KF1 antibody recognized an ~80kDa band, which is similar to the predicted size of CsKF1, in cucumber root, leaf, shoot, and fruit (5 DAA). The anti-KF2 antibody recognized an ~125kDa band in cucumber leaf, shoot, and fruit (0 DAA). The anti-KF3 antibody recognized an ~110kDa band in cucumber root, stem, male flower, female flower, and fruit (2 DAA) (Fig. 8E–G). As expected, each of the three kinesin proteins could be detected more clearly in cucumber fruit cells sampled at the time points at which each gene was up-regulated. An anti-α-tubulin antibody was used as a positive control, and the α-tubulin band was observed in all samples (Fig. 8H). These results indicated that these antibodies specifically recognize CsKF1, CsKF2, and CsKF3 at the predicted sizes in cucumber *in vivo*.

**Fig. 9.**
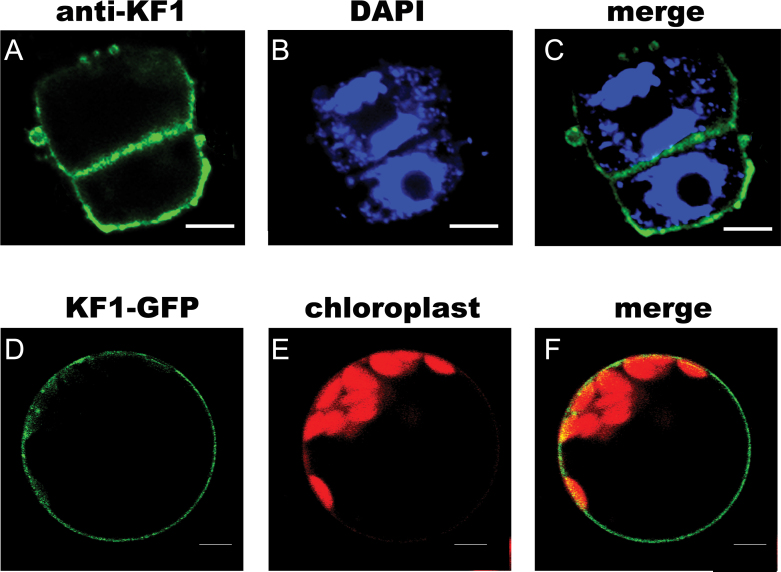
Subcellular localization of CsKF1 in plant cells. (A–C) Immunolocalization of CsKF1 in fruit cells at 5 DAA. The 9930 fruit cells harvested at 5 DAA were stained with the anti-CsKF1 antibody (A; green) and DAPI (B; blue). Strong CsKF1 signals appeared close to the plasma membrane (C). (D–F) In living *Arabidopsis* protoplasts, CsKF1–GFP localized to the plasma membrane. The protoplasts were transiently transformed with the CsKF1–GFP construct (D; green), and the chloroplast autofluorescence was viewed as the red channel (E; red). Strong CsKF1 green signals localized in the plasma membrane (F). Scale bar=5 μm.

Next, these specific antibodies were used to localize CsKF1, CsKF2, and CsKF3 in fruit cells via immunofluorescence. Figure 9A–C shows that CsKF1 was detected at the plasma membrane in fruit cells at 5 DAA. To confirm this result, GFP–KF1 was constructed and transiently expressed in *Arabidopsis* mesophyll protoplasts. It was found that the KF1–GFP signal mainly co-localized with the plasma membrane (Fig. 9D–F). The plasma membrane localization of CsKF1 coincides with the predicted function of CsKF1 in rapid cell enlargement (Table 3)

**Fig. 10.**
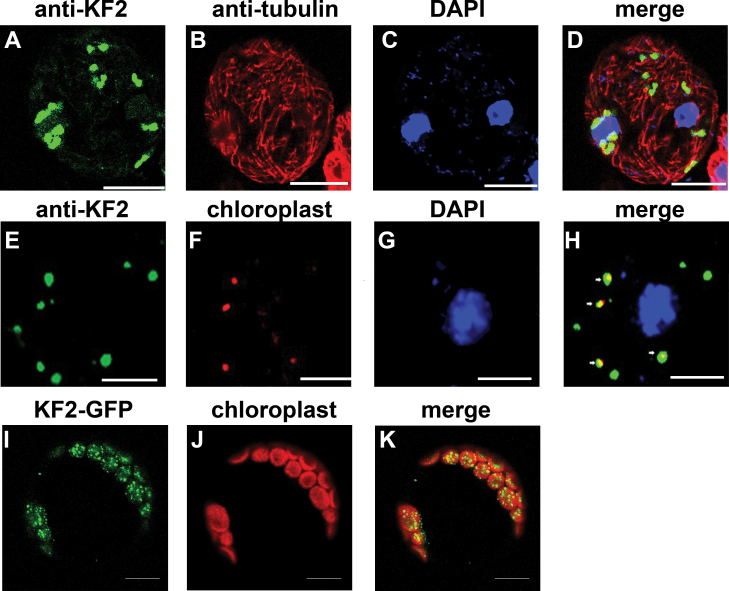
Subcellular localization of CsKF2 in plant cells. (A–D) Triple immunolocalization of CsKF2, tubulin, and the nucleus in fruit cells at 0 DAA. Anti-KF2 antibodies detected particles in a punctate pattern in the cytoplasm in fruit cells at 0 DAA (A; green). Microtubules were immunofluorescently labelled with anti-α-tubulin antibody and TRITC-conjugated goat anti-mouse IgG (B; red). The nucleus was detected by DAPI (C; blue). No CsKF2 signal was associated with microtubules (D; merge). (E–H) Co-localization of CsKF2 with chloroplast in fruit cells at 0 DAA. Anti-KF2 antibodies detected particles in a punctate pattern in the cytoplasm in fruit cells at 0 DAA (E; green). Chloroplast autofluorescence was viewed as the red channel (F; red). The nucleus was detected by DAPI (G; blue). CsKF2 green signals showed co-localization with chloroplasts (H; merge). (I–K) Co-localization of CsKF2–GFP with the chloroplast in *Arabidopsis* protoplasts. The protoplasts were transiently transformed with the CsKF2–GFP construct (I; green), and the chloroplast autofluorescence was viewed through the red channel (J; red). Strong CsKF2 signals appeared in particles in the chloroplasts (K). Scale bar=5 μm.

Interestingly, immunofluorescence labelling showed that anti-KF2 antibodies detected particles (~1 μm) in a punctate pattern in the cytoplasm in fruit cells at 0 DAA. These punctate signals showed no significant co-localization with immunolabeled tubulin (Fig. 10A–D). This result suggests that CsKF2 might work specifically in an organelle.

**Fig. 11.**
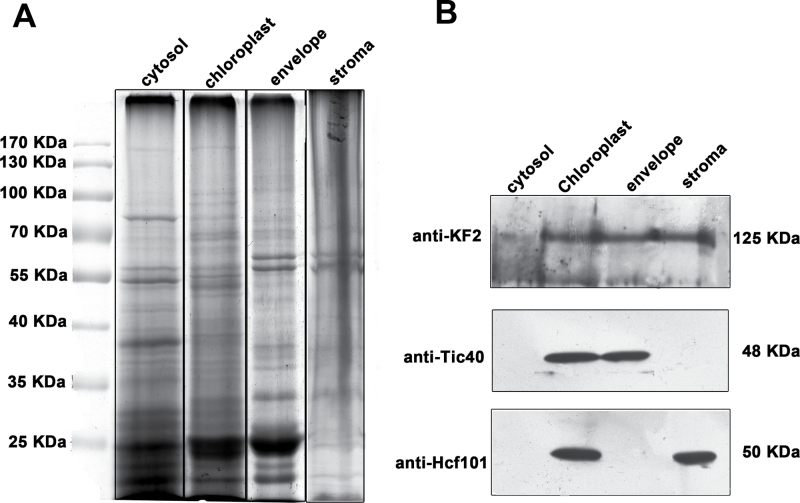
Immunoblotting of chloroplast fractions shows that CsKF2 predominantly localizes to both the membrane and stroma of chloroplasts. (A) SDS–PAGE of protein from the cytosol, chloroplast, chloroplast membrane, and stroma. (B) CsKF2 (detected by anti-CsKF2 antibody) localizes to both the membrane fractions including envelope and thylakoid membranes, and the stroma fraction.

Figure 10E–H showed that immunofluorescence of anti-CsKF2 antibodies merged with chloroplast autofluorescence. To confirm the chloroplast targeting of CsKF2, KF2–GFP was transiently expressed in *Arabidopsis* mesophyll protoplasts. The GFP–KF2 signal was localized in chloroplasts (Fig. 10I–K). To understand the detailed localization of CsKF2 in chloroplasts, its detailed localization was studied in cucumber fruit cells by combining the chloroplast fraction with immunoblotting detection. As shown in Fig. 11, CsKF2 proteins exist predominantly in chloroplasts, in which proteins from both membrane and stroma showed CsKF2 immunoblotting signals. The chloroplast envelope marker Tic40 and stroma marker Hcf101 were used as controls (Fig. 11). To gain insight into how CsKF2 enters the chloroplast, the full-length peptide sequence of CsKF2 was analysed using ChloroP 1.1. The results indicated that a chloroplast transit peptide (cTP; amino acids 1–38) is located at the N-terminus of CsKF2 (Supplementary Table S5 at *JXB* online). To the authors’ knowledge, this is the first reported kinesin-related protein that can enter the chloroplast. It was also found that the CsKF2 fluorescence signals could predominantly be detected in fruit that was harvested at –2 DAA to 2 DAA during rapid cell division (Supplementary Fig. S5). The results suggest that CsKF2 might be involved in the segregation of chloroplast nucleoids and plays a role in chloroplast division during early fruit cell production.

**Fig. 12.**
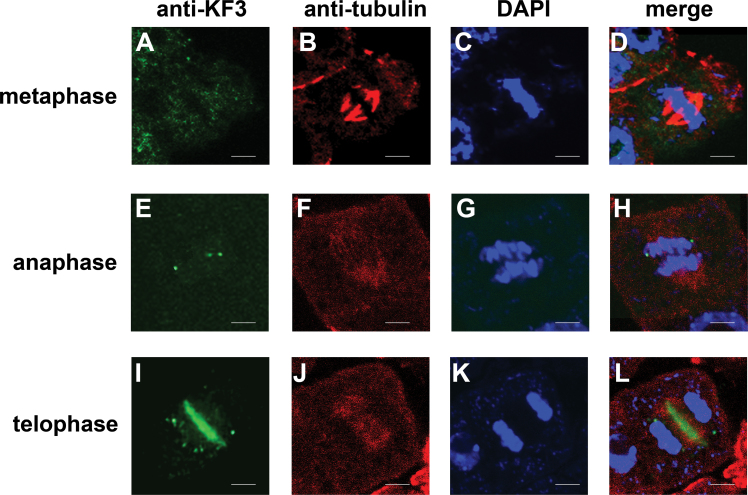
CsKF3 distributes in a cell cycle-dependent manner in cucumber fruit cells. CsKF1 (green), microtubules (red), and the nucleus (stained with DAPI; blue) are shown at different cell division stages. (A–D) In metaphase cells, few CsKF3 punctate signals could be detected by fluorescence microscopy. (E–H) During anaphase, only small CsKF3 punctate signals could be detected by anti-CsKF3 antibodies. (I–L) CsKF3 mainly localized at the midzone of the phragmoplast in telophase cells. Scale bar=5 μm.

Immunofluorescence with the anti-KF3 antibody indicated that CsKF3 localized to the central region of telophase cells in fruit cells at 2 DAA (Fig. 12). This result raised the question of whether the localization of CsKF3 changed dynamically during the cell cycle. Dual localizations of CsKF3 and tubulin were performed. During interphase, CsKF3 was present throughout the cytoplasm and in the nucleus. During the progression of mitosis, no fluorescence signal was detected in the MT pre-prophase band or in MTs on the nuclear envelope (data not shown). In metaphase cells, judged by their spindle MTs, only a few CsKF3 punctate signals could be detected by fluorescence microscopy in the cytoplasm (Fig. 12A–D), and this same staining pattern was observed during anaphase (Fig. 12E–H). When sister chromatids were separated completely during telophase, CsKF3 mainly localized at the midzone of the phragmoplast (Fig. 12I–L). These results indicate that CsKF3 is involved in the function or formation of dynamic phragmoplasts in telophase during rapid division.

## Discussion

The early stages of cucumber fruit development, from initial set through rapid cell division and cell enlargement, are critical determinants of size, yield, and quality (Fu *et al.*, 2008; Ando *et al.*, 2012). Recent studies have indicated that the cytoskeleton has important roles in this process (Ando and Grumet, 2010; Ando *et al.*, 2012). However, there has been little detailed analysis. In the plant cell, the dynamics of MTs have critical roles in the cell cycle, construction of the cell wall, and maintenance of cell morphology. In this study, seven candidate MT-based kinesin genes that are involved in the early exponential growth of cucumber fruit were identified by comparing fruit morphological and cellular analyses using quantitative RT-PCR. Further functional studies showed that CsKF1 localizes to the plasma membrane and may participate in exponential cell expansion; that CsKF2 functions in the chloroplast during the rapid cell division phase, which suggested a role in chloroplast division; and that CsKF3 is involved in the function or formation of dynamic phragmoplasts in telophase during rapid division.

### Early exponential growth of cucumber fruit

Early fruit development is characterized by phases of rapid cell division and expansion (Gillaspy *et al.*, 1993). Previous studies have shown that in cucumber fruit, which develops from an enlarged inferior ovary, cell division occurs most rapidly prior to anthesis and then continues from 0 DAA to 5 DAA (Gillaspy *et al.*, 1993; Robinson and Decker-Walters, 1997; Boonkorkaew *et al.*, 2008). Fruit enlargement begins almost immediately after pollination, with the most rapid increase occurring at ~4–12 DAA (Ando and Grumet, 2010). Cell division and expansion are largely completed by 12–16 DAA, with some variation depending on the cultivar and season (Marcelis and Baan Hofman-Eijer, 1993; Ando and Grumet, 2010; Ando *et al.*, 2012). Here, the morphological and cellular changes during early fruit development were compared in four different cucumber varieties with varied fruit size (Figs 1, 4). The results closely concur with earlier studies. It was found that ~3–5 DAA is the transition from rapid cell division to cell expansion in early cucumber fruit development. Before 3–5 DAA, the cell number increases rapidly, and, following exit from mitotic cell production, an exponential increase in cell size (30-fold) occurs from 5 DAA to 16 DAA (Figs 2, 4). Additionally, the fruits of 9930 and Zhong Nong 27, the varieties with larger fruits, show higher rates of cell division and cell growth than those of the 1972 B-2 and National Pickle fruits, the varieties with smaller fruits (Figs 2, 4). Moreover, the average cell area in 1972 B-2 fruit is almost 20-fold less than the cell area of the other varieties (Fig. 4C), which strongly suggests why 1972 B-2 bears the smallest fruit in the four varieties. To the authors’ knowledge, this study is the first to determine the morphological and cellular changes of fruit in different genotypes of cucumber with varied fruit size, and these changes mirror the basic patterns of early cucumber fruit development.

### Kinesins are involved in early cucumber fruit development

It has been reported that the tubulin gene shows high expression levels in the early phase of cucumber growth, which suggests that tubulin has a critical role in this process (Ando and Grumet, 2010; Ando *et al.*, 2012). The dynamics of MTs are modulated by MT-associated proteins (Olmsted, 1986). Kinesin superfamily proteins are important MT-based motor proteins, with a motor domain that is conserved among all eukaryotic organisms (Li *et al.*, 2012). In addition to the responsibility for unidirectionally transporting various cargos, kinesins also play critical roles in mitosis and cell morphogenesis (Lee and Liu, 2004; Hirokawa *et al.*, 2009; Li *et al.*, 2012; Zhu and Dixit, 2012). It is unclear whether any specific kinesin genes are involved in the rapid cell division and cell enlargement during early cucumber fruit development. In this research, 47 kinesin genes found in 14 kinesin families and one ungrouped family were identified in the cucumber genome (Supplementary Table S3, Supplementary Fig. S1 at *JXB* online). All of these kinesin genes were found to be expressed in various cucumber tissues (Supplementary Fig. S2). All of the kinesin genes except *Cs4g219350* showed a significant change in expression during early fruit development (from –2 DAA to 16 DAA), suggesting important roles for the kinesin gene family in facilitating cell production and growth in cucumber fruit (Fig 3; Supplementary Fig. S3).

Several studies have previously addressed genome-wide changes in gene expression during fruit development, such as in tomatoes and apples (Alba *et al.*, 2004; Lemaire-Chamley *et al.*, 2005; Janssen *et al.*, 2008; Malladi and Johnson, 2011), but there has been little research conducted in cucumbers. Recently, through linking morphological and cellular analyses with 454 pyrosequencing, researchers have studied transcript-level changes occurring in young cucumber fruit at five ages from anthesis through to the end of exponential growth, providing knowledge of early cucumber fruit development (Ando and Grumet, 2010; Ando *et al.*, 2012). In this study, quantitative expression changes of kinesin genes were compared with the changes that occur during cell production and cell expansion during early fruit development in the four cucumber varieties with varied fruit size. It was found that five kinesin genes, *Csa3M062600*, *Csa4M002000*, *Csa6M499030*, *Csa2M250930*, and *Csa5M157410* (*CsKF2*–*CsKF6*), were positively correlated with rapid cell production, and two kinesin genes, *Csa7M446860* and *Csa1M495290* (*CsKF1* and *CsKF7*), showed strong positive correlation with rapid cell expansion (Fig. 5; Tables 1–3). These seven candidate kinesins are distributed across six different kinesin families. Additionally, the *Arabidopsis* homologues of CsKF1, CsKF2, CsKF3, and CsKF7 have unknown functions, while the *Arabidopsis* homologues of CsKF4, CsKF5, and CsKF6 have been reported to work in chloroplast movement, mitochondrial division, and cell mitosis, respectively (Table 3). The results suggest that the cucumber kinesins have various functions in rapid fruit growth.

### Function of novel kinesins CsKF1, CsKF2, and CsKF3 in early cucumber fruit development

Among these seven candidate kinesin genes, *CsKF1*, *CsKF2*, and *CsKF3*, were chosen for further study. The functions of the *Arabidopsis* homologues of these genes are unknown. Expression assays in various cucumber tissues revealed that all the three kinesin genes are more highly enriched in developing fruit (Supplementary Fig. S2 at *JXB* online). Immunoblots with specific polyclonal antibodies against CsKF1, CsKF2, and CsKF3 showed that these proteins could be detected in cucumber fruit sampled at specific times during fruit development (Fig. 8E–G). These results suggest the involvement of these three kinesins during early cucumber fruit development.

Phylogenetic analyses showed that CsKF1 belongs to the kinesin-13 family and is orthologous to AtKINESIN13-B in *Arabidopsis* (Supplementary Fig. S1 at *JXB* online). AtKINESIN13-A and AtKINESIN13-B are a pair of counterparts in *Arabidopsis* (Lee and Liu, 2004). The function of AtKINESIN13-B has not been reported. *AtKinesin13A* mutants have an abnormal trichome phenotype, suggesting the roles of MTs and AtKINESIN13-A in the organization of Golgi stacks, trichome elongation, and cell morphogenesis (Lu *et al.*, 2005). In rice, the kinesin protein SRS3 has an amino acid sequence that resembles that of AtKINESIN13-B in *Arabidopsis* and is involved in regulating cell area and rice seed length (Kitagawa *et al.*, 2010). The present results showed that CsKF1 localizes to the plasma membrane of fruit cells in the rapid expansion stage (Fig. 9) and has a strong positive correlation with rapid cell expansion during early fruit development (Fig. 5A; Tables 1–3). These results provided experimental clues that a novel kinesin CsKF1 played a role in fruit cell expansion in cucumber. It would be interesting to establish the detailed role of CsKF1 by transgenic methods in the future.

Phylogenetic analyses showed that CsKF2, whose homologue in *Arabidopsis* has an unknown function, belongs to the kinesin-12 family (Supplementary Fig. S1at *JXB* online). Biochemical analyses showed that the ATPase activity of His-CsKF2-motor is independent of the concentration of MTs, and this protein binds to MTs in a nucleotide-independent manner, which are uncommon characteristics of kinesins (Fig. 7). Immunolabelling, transient expression of GFP fusion protein, and chloroplast fractionation showed that CsKF2 predominantly localized in the membrane and stroma of chloroplasts in fruit cells (Figs 10, 11). Moreover, a cTP was found in the N-terminus of CsKF2, indicating that CsKF2 functions within the chloroplast (Supplementary Table S5). Previous studies have shown that lipoxygenase (LOX) and hydroperoxide lyase (HPL), which are essential for the production of the cucumber flavour (E,Z)-2,4-nonadienal, are mainly enriched in cucumber chloroplasts (Wardale *et al.*, 1978; Wardale and Ambert, 1980). A recent transcriptome study has indicated that chloroplast-related genes were significantly enriched at 0–4 DAA in early cucumber fruit development, and the expression of these genes declined with age, which is consistent with the peak of chlorophyll content at 4 DAA and the subsequent decrease (Ando *et al.*, 2012). Therefore, chloroplast division during rapid fruit cell division would be important for cucumber fruit development. As is known, the prokaryotic homologue of tubulin, ftsZ, is responsible for plant chloroplast division (Osteryoung *et al.*, 1998; Reski, 2002). In prokaryotes, a kinesin homologue, MukB, interacting with FtsZ, is involved in chromosome partitioning (Niki *et al.*, 1991; Lockhart and Kendrick-Jones, 1998a, *b*). It was inferred that CsKF2 might be involved in chloroplast division, and the partner of CsKF2 might be ftsZ but not tubulin, which could explain the uncommon biochemical properties of CsKF2 that were observed during research with porcine brain tubulins.

CsKF3 belongs to the kinesin-14 family (Supplementary Fig. S1 at *JXB* online). A localization assay showed that CsKF3 localizes mainly at the midzone of the phragmoplast in fruit telophase cells (Fig. 12). Previous studies have revealed several motor proteins in the phragmoplast, such as POK1, POK2, AtPAKRP1, AtPAKRP1L, AtPAKRP2, and GhKCH2. These motor proteins were indicated to be necessary for the functions and formation of the dynamic phragmoplast, such as assisting vesicles and cell wall materials to be correctly oriented and accumulated at the midzone of the phragmoplast (Lee *et al.*, 2001; Pan *et al.*, 2004; Müller *et al.*, 2006; Xu *et al.*, 2009). In the near future, RNA interference lines for *CsKF1*, *CsKF2*, and *CsKF3* should be constructed to observe the relative phenotypes in cucumber fruit. Also understanding the roles of CsKF1, CsKF2, and CsKF3 in MT regulation (or FtsZ regulation) would be also helpful to investigate their fruit-specific functionality.

## Supplementary data

Supplementary data are available at *JXB* online.

Figure S1. Phylogenetic analysis of predicted cucumber kinesins and other representatives of kinesins in plants indicated that all predicted kinesins distribute in 14 families and one plant-specific ungrouped family.

Figure S2. Expression of 47 cucumber kinesin genes during vegetative (root, stem, tendril, true leaf, cotyledon, and hypocotyl) and reproductive development (dry seed, geminated seed, male flower, female flower, ovary, 0 DAA fruit, 2 DAA fruit, and 10 DAA fruit) by using RT-PCR.

Figure S3. Expression profiles of kinesin family genes during early fruit development in cucumber. Quantitative RT-PCR results of the expression of 47 kinesin genes during early 9930 fruit development.

Figure S4. Physiological changes in 1972 B-2, National Pickle, and Zhong Nong 27 during early fruit development.

Figure S5. Protein expression patterns of CsKF2 during cucumber early fruit development.

Table S1. Sequence-specific primers used for construction.

Table S2. Specific primers used for RT-PCR and quantitative RT-PCR.

Table S3. List of predicted kinesin genes in cucumber.

Table S4. Twelve cucumber kinesin genes showed peak expression in the rapid cell division phase (before 3 DAA) or cell expasion phase (after 5 DAA).

Table S5. A predicted chloroplast transit peptide (cTP) in the N-terminus of CsKF2.

Supplementary Data

## References

[CIT0001] Alba R Fei Z Payton P Liu Y Moore SL Debbie P Cohn J D’Ascenzo M Gordon JS Rose JK 2004 ESTs, cDNA microarrays, and gene expression profiling: tools for dissecting plant physiology and development. The Plant Journal 39, 697–714 15315633 10.1111/j.1365-313X.2004.02178.x

[CIT0002] Ambrose JC Cyr R 2007 The kinesin ATK5 functions in early spindle assembly in Arabidopsis. The Plant Cell 19, 226–236 17220198 10.1105/tpc.106.047613 PMC1820958

[CIT0003] Ando K Carr K Grumet R 2012 Transcriptome analyses of early cucumber fruit growth identifies distinct gene modules associated with phases of development. BMC Genomics 13, 518–533 23031452 10.1186/1471-2164-13-518 PMC3477022

[CIT0004] Ando K Grumet R 2010 Transcriptional profiling of rapidly growing cucumber fruit by 454-pyrosequencing analysis. Journal of the American Society for Horticultural Science 135, 291–302

[CIT0005] Bannigan A Scheible W-R Lukowitz W Fagerstrom C Wadsworth P Somerville C Baskin TI 2007 A conserved role for kinesin-5 in plant mitosis. Journal of Cell Science 120, 2819–2827 17652157 10.1242/jcs.009506

[CIT0006] Ben-Cheikh W Perez-Botella J Tadeo FR Talon M Primo-Millo E 1997 Pollination increases gibberellin levels in developing ovaries of seeded varieties of citrus. Plant Physiology 114, 557–564 12223728 10.1104/pp.114.2.557 PMC158336

[CIT0007] Boonkorkaew P Hikosaka S Sugiyama N 2008 Effect of pollination on cell division, cell enlargement, and endogenous hormones in fruit development in a gynoecious cucumber. Scientia Horticulturae 116, 1–7

[CIT0008] Chen C Marcus A Li W Hu Y Calzada J-PV Grossniklaus U Cyr RJ Ma H 2002 The Arabidopsis ATK1 gene is required for spindle morphogenesis in male meiosis. Development 129, 2401–2409 11973272 10.1242/dev.129.10.2401

[CIT0009] Fu F Mao W Shi K Zhou Y Yu J 2010 Spatio-temporal changes in cell division, endoreduplication and expression of cell cycle-related genes in pollinated and plant growth substances-treated ovaries of cucumber. Plant Biology 12, 98–107 20653892 10.1111/j.1438-8677.2009.00203.x

[CIT0010] Fu FQ Mao WH Shi K Zhou YH Asami T Yu JQ 2008 A role of brassinosteroids in early fruit development in cucumber. Journal of Experimental Botany 59, 2299–2308 18515830 10.1093/jxb/ern093 PMC2423651

[CIT0011] Gillaspy G Ben-David H Gruissem W 1993 Fruits: a developmental perspective. The Plant Cell 5, 1439–1451 12271039 10.1105/tpc.5.10.1439 PMC160374

[CIT0012] Hirokawa N Noda Y Tanaka Y Niwa S 2009 Kinesin superfamily motor proteins and intracellular transport. Nature Reviews Molecular Cell Biology 10, 682–696 10.1038/nrm2774 19773780

[CIT0013] Huang S Li R Zhang Z Li L Gu X Fan W Lucas WJ Wang X Xie B Ni P 2009 The genome of the cucumber, Cucumis sativus L. Nature Genetics 41, 1275–1281 19881527 10.1038/ng.475

[CIT0014] Janssen B Thodey K Schaffer R Alba R Balakrishnan L Bishop R Bowen J Crowhurst R Gleave A Ledger S 2008 Global gene expression analysis of apple fruit development from the floral bud to ripe fruit. BMC Plant Biology 8, 16–44 18279528 10.1186/1471-2229-8-16 PMC2287172

[CIT0015] Kitagawa K Kurinami S Oki K Abe Y Ando T Kono I Yano M Kitano H Iwasaki Y 2010 A novel kinesin 13 protein regulating rice seed length. Plant and Cell Physiology 51, 1315–1329 20587735 10.1093/pcp/pcq092

[CIT0016] LeBel D Poirier GG Beaudoin AR 1978 A convenient method for the ATPase assay. Analytical Biochemistry 85, 86–89 147037 10.1016/0003-2697(78)90277-4

[CIT0017] Lee Y-RJ Giang HM Liu B 2001 A novel plant kinesin-related protein specifically associates with the phragmoplast organelles. The Plant Cell 13, 2427–2439 11701879 10.1105/tpc.010225 PMC139462

[CIT0018] Lee Y Liu B 2000 Identification of a phragmoplast-associated kinesin-related protein in higher plants. Current Biology 10, 797–800 10898978 10.1016/s0960-9822(00)00564-9

[CIT0019] Lee Y-RJ Liu B 2004 Cytoskeletal motors in Arabidopsis. Sixty-one kinesins and seventeen myosins. Plant Physiology 136, 3877–3883 15591445 10.1104/pp.104.052621 PMC535821

[CIT0020] Lemaire-Chamley M Petit J Garcia V Just D Baldet P Germain V Fagard M Mouassite M Cheniclet C Rothan C 2005 Changes in transcriptional profiles are associated with early fruit tissue specialization in tomato. Plant Physiology 139, 750–769 16183847 10.1104/pp.105.063719 PMC1255993

[CIT0021] Li J Jiang J Qian Q Xu Y Zhang C Xiao J Du C Luo W Zou G Chen M 2011 Mutation of rice BC12/GDD1, which encodes a kinesin-like protein that binds to a GA biosynthesis gene promoter, leads to dwarfism with impaired cell elongation. The Plant Cell 23, 628–640 21325138 10.1105/tpc.110.081901 PMC3077781

[CIT0022] Li J Xu Y Chong K 2012 The novel functions of kinesin motor proteins in plants. Protoplasma 249, 95–100 10.1007/s00709-011-0357-3 PMC3389602 22167300

[CIT0023] Livak KJ Schmittgen TD 2001 Analysis of relative gene expression data using real-time quantitative PCR and the 2^−ΔΔ*C*T^ method. Methods 25, 402–408 11846609 10.1006/meth.2001.1262

[CIT0024] Lockhart A Kendrick-Jones J 1998a Interaction of the N-terminal domain of MukB with the bacterial tubulin homologue FtsZ. FEBS Letters 430, 278–282 9688555 10.1016/s0014-5793(98)00677-2

[CIT0025] Lockhart A Kendrick-Jones J 1998b Nucleotide-dependent interaction of the N-terminal domain of MukB with microtubules. Journal of Structural Biology 124, 303–310 10049813 10.1006/jsbi.1998.4056

[CIT0026] Lu L Lee Y-RJ Pan R Maloof JN Liu B 2005 An internal motor kinesin is associated with the Golgi apparatus and plays a role in trichome morphogenesis in Arabidopsis. Molecular Biology of the Cell 16, 811–823 15574882 10.1091/mbc.E04-05-0400 PMC545913

[CIT0027] Malladi A Johnson LK 2011 Expression profiling of cell cycle genes reveals key facilitators of cell production during carpel development, fruit set, and fruit growth in apple (Malus×domestica Borkh.). Journal of Experimental Botany 62, 205–219 20732881 10.1093/jxb/erq258 PMC2993910

[CIT0028] Marcelis L Baan Hofman-Eijer LR 1993 Cell division and expansion in the cucumber fruit. Journal of Horticultural Science 68: 665–671

[CIT0029] Mounet F Moing A Garcia V Petit J Maucourt M Deborde C Bernillon S Le Gall G Colquhoun I Defernez M 2009 Gene and metabolite regulatory network analysis of early developing fruit tissues highlights new candidate genes for the control of tomato fruit composition and development. Plant Physiology 149, 1505–1528 19144766 10.1104/pp.108.133967 PMC2649409

[CIT0030] Müller S Han S Smith LG 2006 Two kinesins are involved in the spatial control of cytokinesis in *Arabidopsis thaliana* . Current Biology 16, 888–894 16682350 10.1016/j.cub.2006.03.034

[CIT0031] Niki H Jaffé A Imamura R Ogura T Hiraga S 1991 The new gene mukB codes for a 177 kd protein with coiled-coil domains involved in chromosome partitioning of *E. coli* . EMBO Journal 10, 183–193 1989883 10.1002/j.1460-2075.1991.tb07935.x PMC452628

[CIT0032] Olmsted J 1986 Microtubule-associated proteins. Annual Review of Cell Biology 2, 421–457 10.1146/annurev.cb.02.110186.002225 3548773

[CIT0033] Osteryoung KW Stokes KD Rutherford SM Percival AL Lee WY 1998 Chloroplast division in higher plants requires members of two functionally divergent gene families with homology to bacterial ftsZ. The Plant Cell 10, 1991–2004 9836740 10.1105/tpc.10.12.1991 PMC143974

[CIT0034] Pan R Lee Y-RJ Liu B 2004 Localization of two homologous Arabidopsis kinesin-related proteins in the phragmoplast. Planta 220, 156–164 15258761 10.1007/s00425-004-1324-4

[CIT0035] Reski R 2002 Rings and networks: the amazing complexity of FtsZ in chloroplasts. Trends in Plant Science 7, 103–105 11906832 10.1016/s1360-1385(02)02232-x

[CIT0036] Robinson RW Decker-Walters D 1997 Cucurbits. Wallingford, UK: CABI Publishers

[CIT0037] Sasabe M Boudolf V De Veylder L Inzé D Genschik P Machida Y 2011 Phosphorylation of a mitotic kinesin-like protein and a MAPKKK by cyclin-dependent kinases (CDKs) is involved in the transition to cytokinesis in plants. Proceedings of the National Academy of Sciences, USA 108, 17844–17849 10.1073/pnas.1110174108 PMC3203811 22006334

[CIT0038] Schlosser J Olsson N Weis M Reid K Peng F Lund S Bowen P 2008 Cellular expansion and gene expression in the developing grape (Vitis vinifera L.). Protoplasma 232, 255–265 18421552 10.1007/s00709-008-0280-9

[CIT0039] Vale RD Fletterick RJ 1997 The design plan of kinesin motors. Annual Review of Cell and Developmental Biology 13, 745–777 10.1146/annurev.cellbio.13.1.745 9442886

[CIT0040] Vanstraelen M Inzé D Geelen D 2006 Mitosis-specific kinesins in *Arabidopsis* . Trends in Plant Science 11, 167–175 16530461 10.1016/j.tplants.2006.02.004

[CIT0041] Wardale DA Ambert EA 1980 Lipoxygenase from cucumber fruit: localization and properties. Phytochemistry 19, 1013–1016

[CIT0042] Wardale DA Lambert EA Galliard T 1978 Localization of fatty acid hydroperoxide cleavage activity in membranes of cucumber fruit. Phytochemistry 17, 205–212

[CIT0043] Xu T Qu Z Yang X Qin X Xiong J Wang Y Ren D Liu G 2009 A cotton kinesin GhKCH2 interacts with both microtubules and microfilaments. Biochemical Journal 421, 171–180 19416090 10.1042/BJ20082020

[CIT0044] Yoo S-D Cho Y-H Sheen J 2007 Arabidopsis mesophyll protoplasts: a versatile cell system for transient gene expression analysis. Nature Protocols 2, 1565–1572 10.1038/nprot.2007.199 17585298

[CIT0045] Yu JQ Li Y Qian YR Zhu ZJ 2001 Cell division and cell enlargement in fruit of Lagenaria leucantha as influenced by pollination and plant growth substances. Plant Growth Regulation 33, 117–122

[CIT0046] Zhou S Wang Y Li W Zhao Z Ren Y Wang Y Gu S Lin Q Wang D Jiang L 2011 Pollen semi-sterility1 encodes a kinesin-1-like protein important for male meiosis, anther dehiscence, and fertility in rice. The Plant Cell 23, 111–129 21282525 10.1105/tpc.109.073692 PMC3051251

[CIT0047] Zhu C Dixit R 2012 Functions of the Arabidopsis kinesin superfamily of microtubule-based motor proteins. Protoplasma 249, 887–899 22038119 10.1007/s00709-011-0343-9

