# Supplementary material for: Characterization and expression profiling of cucumber kinesin genes during early fruit development: revealing the roles of kinesins in exponential cell production and enlargement in cucumber fruit
Source: J Exp Bot. 2013 Sep 10;64(14):4541–57. doi: 10.1093/jxb/ert269 (PMC3808332; doi:10.1093/jxb/ert269)
Supplement: Supplementary Data [file supp_64_14_4541__index.html]

Characterization and expression profiling of cucumber kinesin genes during early fruit development: revealing the roles of kinesins in exponential cell production and enlargement in cucumber fruit — Characterization and expression profiling of cucumber kinesin genes during early fruit development: revealing the roles of kinesins in exponential cell production and enlargement in cucumber fruit — Supplementary Data 

# Characterization and expression profiling of cucumber kinesin genes during early fruit development: revealing the roles of kinesins in exponential cell production and enlargement in cucumber fruit

## Supplementary Data

Data files

**Files in this Data Supplement:**

- Supplementary Data - Supplementary Data
