# Supplementary material for: Characterization and expression profiling of cucumber kinesin genes during early fruit development: revealing the roles of kinesins in exponential cell production and enlargement in cucumber fruit
Source: J Exp Bot. 2013 Sep 10;64(14):4541–57. doi: 10.1093/jxb/ert269 (PMC3808332; doi:10.1093/jxb/ert269)
Supplement: Supplementary Data [file supp_ert269_jexbot101881_file001.pdf]

Supplementary Data

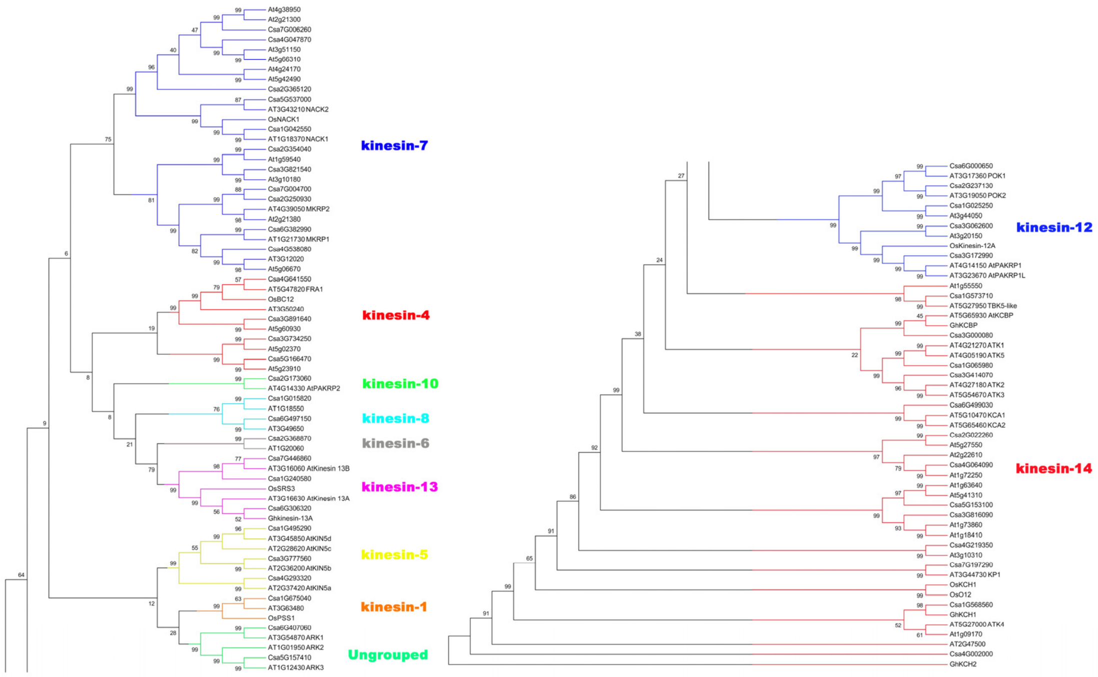

Fig. S1 Phylogenetic analysis of predicted cucumber kinesins and other representatives of kinesins in plant indicated that all predicted kinesins distribute in 14 families and one plant-specific ungrouped family.

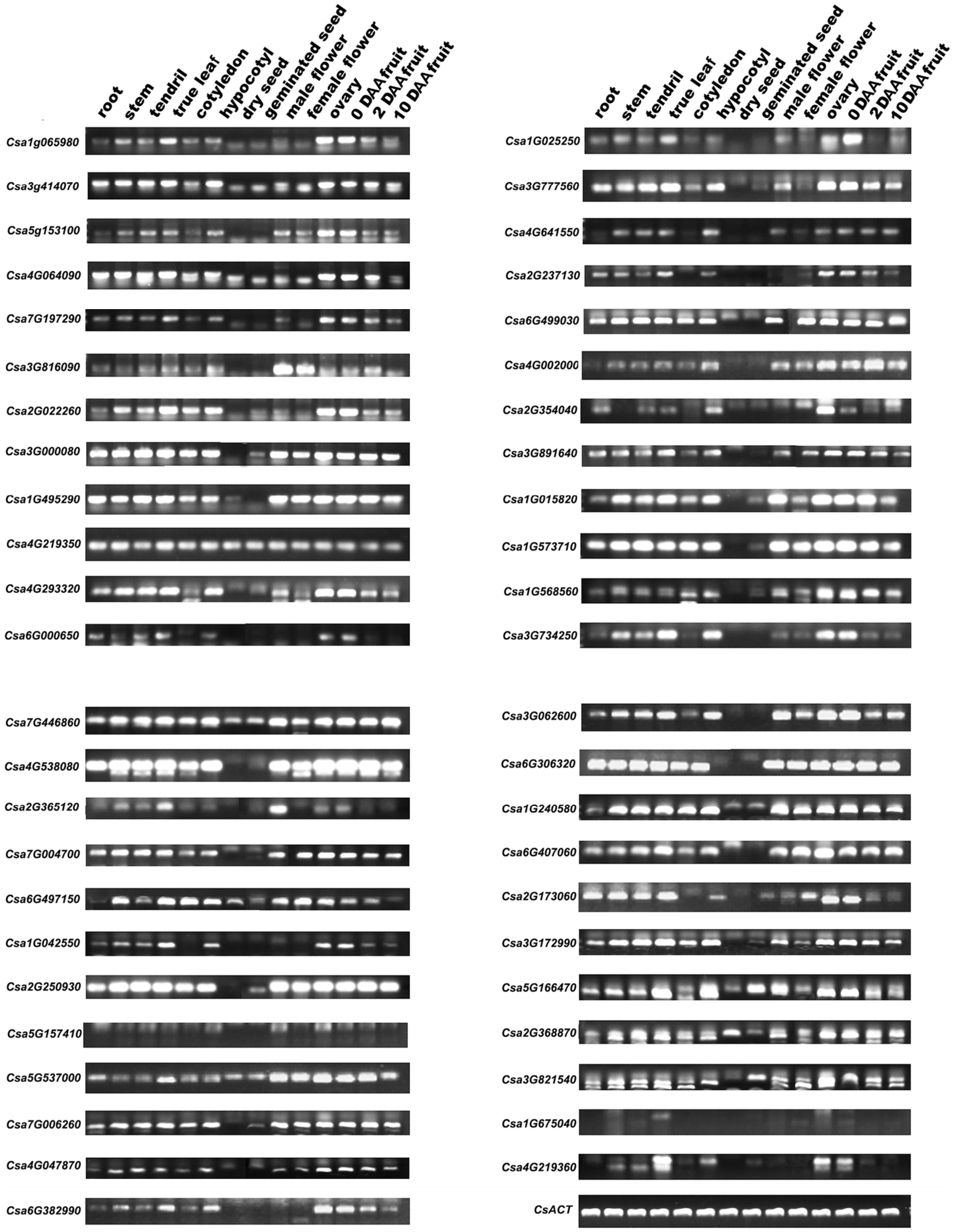

Fig. S2 Expression of forty-seven cucumber kinesin genes during vegetative (root, stem, tendril, true leaf, cotyledon and hypocotyl) and reproductive development (dry seed, geminated seed, male flower, female flower, ovary, 0 DAA fruit, 2 DAA fruit and 10 DAA fruit) by using RT-PCR.

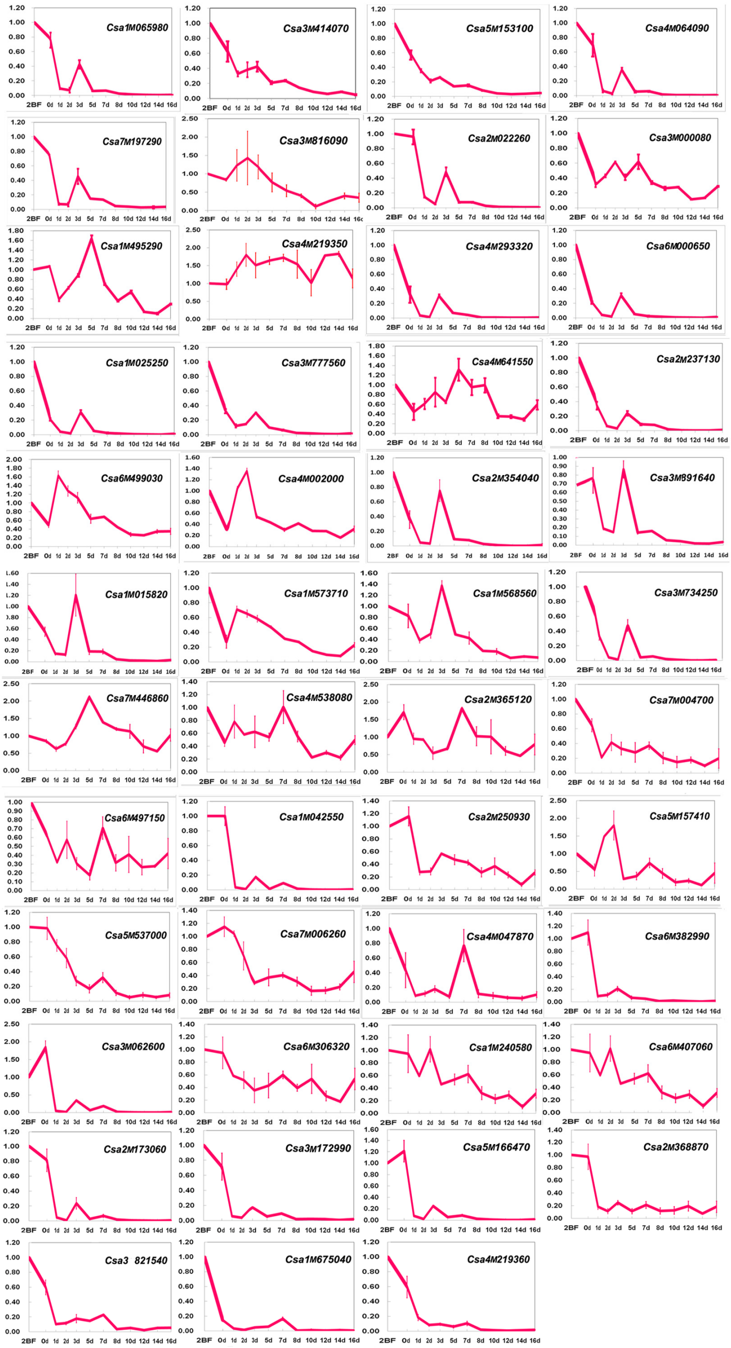

Fig. S3 Expression profiles of kinesin family genes during early fruit development in cucumber. Quantitative RT-PCR results of the expression of 47 kinesin genes during early 9930 fruit development. These data represent the fold changes in expression relative to the expression at -2 DAA. The error bars represent the standard error of the means (n=3).

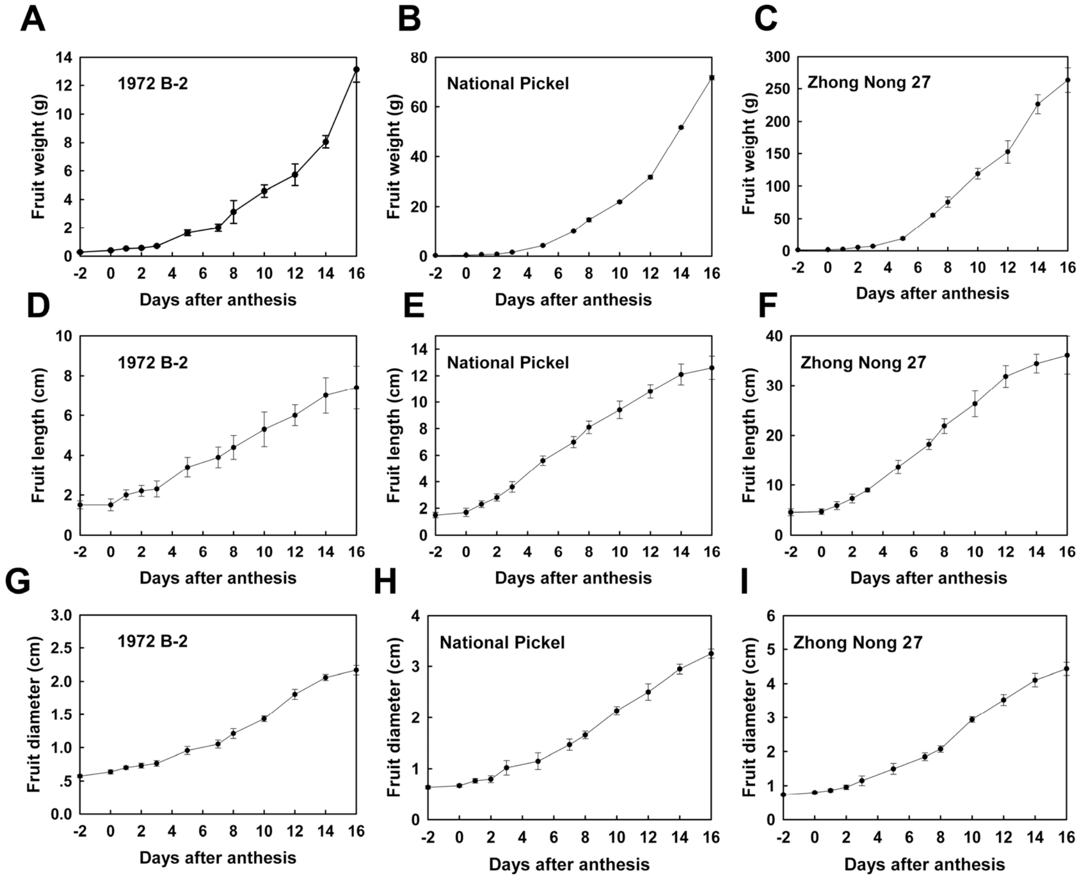

Fig. S4 Physiological changes in 1972 B-2, National Pickle and Zhong Nong 27 during early fruit development. Increase in weight of 1972 B-2 (A), National Pickle (B) and Zhong Nong 27 (C) fruit during early fruit development. Increase in length of 1972 B-2 (D), National Pickle (E) and Zhong Nong 27 (F) fruit during early fruit development. Increase in diameter of 1972 B-2 (G), National Pickle (H) and Zhong Nong 27 (I) fruit during early fruit development.

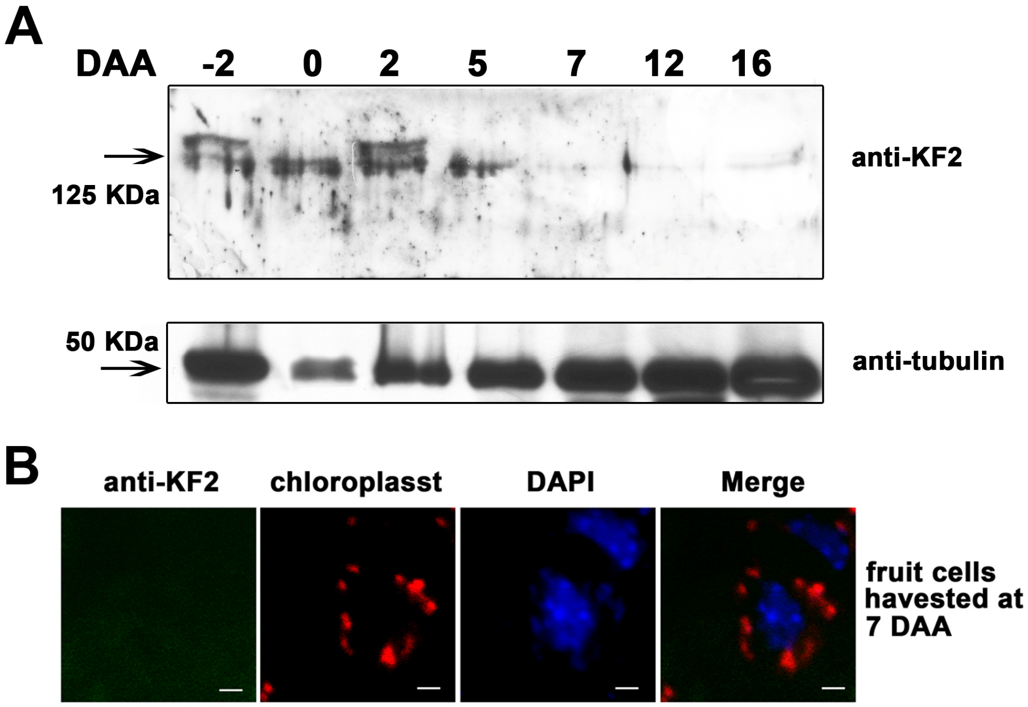

Fig. S5 Protein expression patterns of CsKF2 during cucumber early fruit development. (A) CsKF2 proteins exist predominantly in early stage (before 5 DAA) of cucumber fruit development. (B) It was failed to immunolable CsKF2 in fruit cells havested at 7 DAA.

Table S1 Sequence-specific primers used for construction.

F represents forward; R represents reverse.

**Construct**	**Primer sequence (5' to 3')**	Restriction site
KF1-N	F: GAATTCATGAATGGAATGGGGAGA; R: GTCGACTCAAGAGTTTGCCCTCTCT	EcoR I Sal I
KF1-M	F: GGATCCATGGTTGCCAAGATCAAAG; R: CTCGAGTCAGACATTATTTCCTTTTGAAAG	BamH I Xho I
KF2-C	F: GGTACCATGGTTCTTGGACATGCTAG; R: CTCGAGTTAATAGTGCTGCTCTTGA	Kpn I Xho I
KF2-M	F: GGTACCATGGATCCACCCATCAAG; R: CTCGAGTCATTCATTTATGATTGGCTGA	Kpn I Xho I
KF3-M	F: GAATTCATGAAGGGTAGCATAAGAGTTT; R: GTCGACTCAGTTCACTCGAGCAG	EcoR I Sal I
KF3-C	F: GGTACCATGCAAATCGAGAATCCA; R: CTCGAGTTATTGTTTTCTATTTCCAGTTC	Kpn I Xho I
KF1	For transient expression<br>F: ACTAGTATGAATGGAATGGGGAGAC; B: GTCGACGTCAGATGAAGATATAAGTACA	Spe I Sal I
KF2	For transient expression<br>F: GTCGACATGAAGTCGAATCCAACAGG; B: GGTACCATAGTGCTGCTCTTGATATATAGC	Sal I Kpn I

Table S2 Specific primers used for RT-PCR and quantitative RT-PCR.

F represents forward; R represents reverse.

Genes	Primers
Csa1M065980	F: TCTTCTCCAGCCATGCCTAG
	R: AATCTACAGGTCGCATGGTTG
Csa3M414070	F: CTATCTTCTTCAGCCATGTCTCG
	R: GTCGTGTATTGGTTAAACGCC
Csa5M153100	F: TCCAGAAAAAACCAATCCACC
	R: CACCAATTGGAGAGCTTTTTTG
Csa4M064090	F: GACAAGGTCAAAGAACTTGAAGC
	R: GACTACCTAACGGTCGACTTGC
Csa7M197290	F: GAACCACCACCAAGAATGACA
	R: GGTATTACCAAAAATTTCCCGC
Csa3M816090	F: GTTGGAAAAGCCCAGATCTACA
	R: GCCATCTTCTACCAGACTTGAGTC
Csa2M022260	F: CAAGAAAAGGTTCGAGATATCGAG
	R: GGCGGTTTCTTCTCTGGAAT
Csa3M000080	F: CTCATGTATGCATCAAGAGTTCG
	R: CTTTAGTATGTCGCTCGTTTTGG
Csa1M495290	F: GAATACACGGTTGATGAACCATC
	R: CCAGCTAAATGCTTTACATCTCC
Csa4M219350	F: GCTCTGAGAAGAAACAATATTACAGG
	R: TCTAGATTTTCCTGGCCCTGT
Csa4M293320	F: GATTATCTGGTCGATCAGCATTC
	R: GAACTTTTCCTTCTATCAAACCCC
Csa6M000650	F: GGGATACTGAAGACGGAAAACA
	R: TTTGAGTTAGCCAACCTCATTG
Csa1M025250	F: GCTGAAAAGGCATGTAGATGAAT

	R: ATGGCATCGACCATCGTC
Csa3M777560	F: CAATATGGGGACTATGAGCCA
	R: GATGGTTTCTCTTTGCCATTACTC
Csa4M641550	F: TCGTGTAGGAGTTGAATGGTACC
	R: TGCCTGATCCATTCTGAAAGT
Csa2M237130	F: CGTTTTAAAGGCAGCTGGAG
	R: TCTTTGAACTCTTCGGTGAAGG
Csa6M499030	F: TCGTCAGATAGTTGAGTTTACTCCA
	R: CACCACACTTGATGATAAAAGGAG
Csa4M002000	F: GCCCAGAGTTAAGCAAGAACTTC
	R: TGTTTTCTATTTCCAGTTCTGCG
Csa2M354040	F: TCTTCTCTCCAGAGTGCCAAA
	R: GCTCCCACGAAGTACGTCATAT
Csa3M891640	F: CACATTGGTAAAATCAAAGGCA
	R: AACGGGATGTTGACAACCTC
Csa1M015820	F: CAGAACTAAGGTTGCAGAAGCA
	R: CAGGGATTGTGCCTGTAATGT
Csa1M573710	F: CTACAGGAGGACTTGAAGAAGCA
	R: CACGTGGATGATGATCGCTA
Csa1M568560	F: CGGAGGCTCAAAAGCAAG
	R: ACCATAGCTTTGTCGTCCCA
Csa3M734250	F: TTGAATACAGCAAGCAGAGAGG
	R: CTCTTGCAGCTTTGCTGAATAAG
Csa7M446860	F: CTGTTGCAGGAAGAGGAAGATC
	R: ATAGATTGCTGCAGCCTTTTG
Csa4M538080	F: GATTAGCACAGGAGGTTGATGAA
	R: CAACTTCTTCTGATAAGGCTTTGAG
Csa2M365120	F: CAAACTTCAAGCTTGAAGGCTC
	R: TCTCGACCATAACATTCGTGC

Csa7M004700	F: TAAAGGCACGGATGCAAGA
	R: CGGAACAAGCAAGTGAGCA
Csa6M497150	F: CTGGAATAGAGGAAGGAAGCC
	R: CCACCATGAAAAGAATGACTTGT
Csa1M042550	F: TCGCTCGTCAAGCATGAG
	R: GGATTCGTCCAGAGCTTGTTTA
Csa2M250930	F: CTCAAGGCAAAGATGCAGG
	R: CGGAACAAGCAAGAGAACAAG
Csa5M157410	F: TAGCACAGCATGAAGTGAACG
	R: CCGTAATCGTCTCATTTCAGC
Csa5M537000	F: CTGTTTATGCAAGTGTCAAGGCT
	R: GGATCTGTCCATAGCTTGTTCAC
Csa7M006260	F: ACGGTCAGGTTCTGGAGGAT
	R: TTGAGACGTTGCAAGTTTGC
Csa4M047870	F: CTGACACAAGCATTGAGTTTGA
	R: TTCCACACAAGATGAGCCAA
Csa6M382990	F: TCCCAAGTTATGCAGGCTG
	R: GGTAACTTCTTCAGCTAAGTTTTGG
Csa3M062600	F: CATCAGTTGCAGAGGAGAGTTTTA
	B: TCGTCATGATACAGTGGTTCAAG
Csa6M306320	F: GGAGATAGTGCGAGAAGAAATGA
	B: CTTTTTCGGCTCAGTATCTCTTG
Csa1M240580	F: CGACATTGTTAGAGAGGAGATGAAC
	B: GTGCCACCAAAACATTGTATTC
Csa6M407060	F: GCGCAAAATGAGGAAAATG
	B: ATGAGCCTGAACCTGAGCC
Csa2M173060	F: TCAGACCTGACGAACCAGAAT
	B: GTGCCCGGTAGCTTGTTCT
Csa3M172990	F: CTGCTCAGATGCGAGCAAT

	B: TGCTTTCATCATCATAAGCAGG
Csa5M166470	F: CACAGCTAGCAAGGAAGAATTG
	B: GCTCACCAGCTTCTTTTTTCAT
Csa2M368870	F: ACCAAAAAGGAGACTTCTGCC
	B: ATGCGAATGAGGGAGATGTT
Csa3M821540	F: GATGCATGATGATGAGGTTCA
	B: TTCTTCAAGTCCTTCGTGGC
Csa1M675040	F: ATAGTATGGAGGGGCCTGGT
	B: GCTGGAAAATAATCATACCAGTAGC
Csa4M219360	F: CACACCATGAATGGTCCATCT
	B: AGAAGGTCTCGGACTTGTTCATT
Csa6a484600 (*CsACTIN*)	F: TCCACGAGACTACCTACAACTC
	B: GCTCATACGGTCAGCGAT

Table S3. List of predicted kinesin genes in cucumber

**Gene ID**	**predicted protein length (aa)**	***Arabidopsis* homologue**	**Gene ID**	**predicted protein length (aa)**	***Arabidopsis* homologue**
Csa1M065980	798	ATK1	*Csa7M446860*	685	KINESIN-13B
Csa3M414070	752	ATK3	*Csa4M538080*	765	AT5G06670
Csa5M153100	1119	AT1G63640	*Csa2M365120*	956	AT2G21300
Csa4M064090	1118	AT1G72250	*Csa7M004700*	1068	MKRP2
Csa7M197290	1195	KP1	*Csa6M497150*	698	AT3G49650
Csa3M816090	1374	AT1G18410	*Csa1M042550*	960	HINKEL
Csa2M022260	794	AT5G27550	*Csa2M250930*	1083	MKRP2
Csa3M000080	1265	ZWI	*Csa5M157410*	914	ARK3
Csa1M495290	1051	AT3G45850	*Csa5M537000*	938	TETRASPORE
Csa4M219350	554	AT3G10310	*Csa7M006260*	1027	AT2G21300
Csa4M293320	1049	AT2G37420	*Csa4M047870*	993	AT3G51150

Csa6M000650	2224	POK1	*Csa6M382990*	1091	MKRP1
Csa1M025250	1393	AT3G44050	*Csa3M062600*	1139	AT3G20150
Csa3M777560	1009	AT2G36200	*Csa6M306320*	805	KINESIN-13A
Csa4M641550	1050	FRA1	*Csa1M240580*	715	AT3G16060
Csa2M237130	2916	POK2	*Csa6M407060*	1060	ARK1
Csa6M499030	1276	KCA1/KAC1	*Csa2M173060*	843	PAKRP2
Csa4M002000	1022	AT2G47500	*Csa3M172990*	1342	KINESIN-12B
Csa2M354040	842	ZCF125	*Csa5M166470*	712	AT5G23910
Csa3M891640	1274	AT5G60930	*Csa2M368870*	870	AT1G20060
Csa1M015820	759	AT1G18550	*Csa3M821540*	1251	AT3G10180
Csa1M573710	631	AT5G27950	*Csa1M675040*	171	AT3G63480
Csa1M568560	1094	ATK4	*Csa4M219360*	537	ATK4
Csa3M734250	622	AT5G02370			

47 kinesin genes were identified in cucumber genome by using the conserved motor domains of *Arabidopsis*, rice and mouse as queries to blast in cucumber genome database, and all these kinesins have corresponding homologues in *Arabidopsis*.

Table S4. Twelve cucumber kinesin genes showed the peak expressions in the rapid cell division phase (before 3 DAA) or cell expasion phase (after 5 DAA)

peak expressions are induced in rapid cell division phase (before 3 DAA)		peak expressions are induced in rapid cell expansion phase (after 5 DAA)	
Gene ID	*Arabidopsis* homologue	Gene ID	*Arabidopsis* homologue
Csa3M816090	AT1G18410	*Csa1M495290*	AT3G45850
Csa6M499030	KCA1/KAC1	*Csa7M446860*	KINESIN-13B
Csa4M002000	AT2G47500		
Csa1M568560	ATK4		
Csa1M042550	HINKEL		

Csa2M250930	MKRP2
Csa5M157410	ARK3
Csa6M382990	MKRP1
Csa3M062600	AT3G20150
Csa5M166470	AT5G23910

Table S5. A predicted chloroplast transit peptides (cTP) in N-terminus of CsKF2

Name	Length	Score	chloroplast transit peptides (cTP)	Score for the suggested cleavage site	cTP- length
CsKF2	1139 aa	0.569	Yes	-0.412	1 aa-38 aa

A chloroplast transit peptides (cTP) locating in CsKF2-N terminus (1 aa – 38 aa) was predicted by ChloroP 1.1 Server at http://www.cbs.dtu.dk/services/ChloroP/.
